# A Finite Element Study of Bimodulus Materials with 2D Constitutive Relations in Non-Principal Stress Directions

**DOI:** 10.3390/ma18225126

**Published:** 2025-11-11

**Authors:** Chao Dong, Fei Wang, Tongtong Wang, Long Zhao, Penghui Qian, Mingfeng Li, Zhenglong Dai, Shan Zeng

**Affiliations:** 1 AECC Shenyang Engine Research Institute, Shenyang 110015, China; daweishengli@163.com; 2 National Key Laboratory of Marine Engine Science and Technology, Shanghai 201108, China; 3 School of Power and Energy Engineering, Nanchang Hangkong University, Nanchang 330063, China; 70747@nchu.edu.cn; 4 School of Aeronautics and Astronautics, Nanchang Hangkong University, Nanchang 330063, China

**Keywords:** bimodulus elasticity, constitutive equation, finite element analysis, structural mechanics, material behavior

## Abstract

This paper extends the application of bimodulus elasticity theory by formulating a constitutive relation applicable to non-principal stress directions, building upon the established framework based on principal stresses. The paper develops four full-scale finite element models—the 3-node triangular, the 4-node quadrilateral, the 6-node triangular, and the 8-node quadrilateral elements—with the latter two showcasing higher precision in complex stress simulations. This formulation enables a more detailed analysis of material behavior under varying stress states. An effective iterative solution approach is introduced to address the nonlinearity of bimodulus materials, ensuring model convergence and reliability. The accuracy of the model has been verified through rigorous ANSYS 2022 R1 simulations, and the solution results have been compared with those in the existing literature, emphasizing the importance of the tension-to-compression modulus ratio in determining structural displacement and stress distribution. The developed models and methods provide useful numerical tools for the analysis and design of structures incorporating bimodulus materials.

## 1. Introduction

Accurate prediction and understanding of material mechanical behavior are crucial for engineering design and scientific research. Traditional elasticity theory, while successful in many aspects, assumes homogeneity and isotropy, which limits its applicability in describing anisotropic materials. For bimodulus materials, which exhibit different moduli under tension and compression, more sophisticated theories are necessary to accurately capture their complex mechanical responses.

Bimodulus characteristics are evident in a variety of engineering materials, such as concrete, bamboo, graphite, new polymer materials, metal alloys, composite materials, and biomedical materials, which exhibit different mechanical properties under tension and compression [1,2,3,4,5,6,7]. The bimodulus features of these materials are crucial for engineering design and material science, as they directly affect the response and behavior of structures. For instance, Bert’s research [8] on fiber-reinforced composites indicates that these materials have different moduli under tension and compression. Destrade et al.’s [9] study on rubber reveals its nonlinear behavior under various stress states. Ma-Magdalena Pastor-Artigues et al. [10] employed different testing methods to characterize the bimodular properties of PLA material. Jones [11] further emphasizes the importance of considering different moduli in material modeling and structural analysis. Ambartsumyan [12]’s theory of elasticity provides a theoretical foundation for functionally graded materials, while He et al. [13] offer analytical solutions for functionally graded thin plates with distinct tension and compression moduli. These examples highlight the importance and necessity of research on bimodulus materials.

The development of bimodulus theory has been an essential supplement to traditional elasticity theory. Its genesis is rooted in the foundational work of Timoshenko [14], who introduced the concept to address the varying mechanical responses of materials under tension and compression. This concept was further developed by Jones [15], who examined the apparent flexural modulus and strength of materials with multiple moduli, providing a more nuanced understanding of material behavior. Bert [8] contributed significantly by developing models specifically tailored for fibrous composites with different properties in tension and compression. His and others’ research [16] on composite stacked structures helped to advance the understanding of bimodulus materials in practical applications. Ambartsumyan [17] further advanced the field by proposing a model for isotropic materials based on the positive–negative sign criterion of principal stresses. This model introduced a bilinear relation with discontinuous tangents at the origin, which has been widely adopted for its applicability to continuous, homogeneous, and isotropic materials. Reddy and Chao [18], Zinno and Greco [19], and others have contributed to the study of nonlinear bending and damage evolution in bimodulus materials, expanding the theoretical and practical knowledge of these materials’ behavior under various conditions. Chen et al. [20] conducted research on the free vibration and dynamic stability of asymmetric bimodulus thick annular plates, investigating the dynamic stability of annular plates subjected to a combination of pure dynamic bending and uniform dynamic tensile stress. The application of bimodulus theory to functionally graded materials has been explored by Li et al. [21] and He et al. [13,22], providing analytical solutions for beams and plates with different moduli in tension and compression. Gao and colleagues [23,24,25] have conducted analyses of structures such as canopies, plates, and thin-shell structures using the bimodulus theory, demonstrating its versatility in different structural applications. Yufan Yan et al. [26] developed a theoretical model that uses a logarithmic function to describe the gradient distribution of tensile and compressive moduli across the cross-section and validated the model’s effectiveness in predicting neutral axis position and modulus degradation through finite element simulations. Ye et al. [27,28] and Yao et al. [29] have made progress in the elasticity theory with different moduli and their relation to finite element methods, as well as the nonlinear mechanical behavior of combined members with different moduli. Rong et al. [30] developed an efficient topology optimization method for bimodulus structures considering displacement constraints. Latorre and Montáns [31] addressed the issue of lack of convergence when dealing with bilinear materials using widely employed methods based on the Ambartsumyan theory. They presented a generalized framework based on hyperelasticity to enhance convergence and achieved the expected results. Du et al. [32] established a new computational framework for materials with different mechanical responses in tension and compression, addressing the convergence difficulties of traditional iterative methods. Rebecca Grazzini et al. [33] conducted uniaxial tension and compression tests, three-point and four-point bending tests, as well as fracture energy tests on bimodular materials. Pan et al. [34] performed fracture analysis for bimodulus materials, while He et al. [35] applied the perturbation–variation method to large deformation bimodulus cylindrical shells, providing a comparative study of bending theory and membrane theory. Ren et al. [36] conducted research on the thermoelastic response of bimodulus materials in response to the wrinkling problem of bimodulus membranes under mechanical loads and temperature variations. While the bimodulus model has gained acceptance due to its empirical alignment and computational feasibility, it remains anchored in the principal stress framework, which can limit its application when principal stress directions deviate from predefined axes. For instance, in the bending analysis of beams, models based solely on the principal stress framework often fail to satisfactorily simulate the internal stress distribution and accurately capture the shift in the neutral layer, leading to inaccurate calculations of equivalent bending stiffness [21,22]. Li et al. [21] and He et al. [22] demonstrated that for functionally graded beams with distinct tension–compression moduli, the principal stress-based model could not adequately predict the neutral axis position, resulting in significant deviations in stress distribution. Similarly, Ye et al. [28] and Zhao et al. [37] highlighted through numerical and analytical studies that the equivalent stiffness and stress state in beam–column members under bending were miscalculated when the critical effect of neutral layer offset was overlooked by the principal stress criterion.

To address the engineering application issues of bimodulus materials, the finite element method (FEM) has been widely used for numerical simulation. In the simulation of bimodulus materials, FEM allows for detailed analysis of complex geometries and loading conditions, providing a powerful tool for predicting material behavior under various stress states. This method is particularly important given that the elastic mechanics problem for materials with different moduli in tension and compression is essentially a nonlinear (or bilinear) problem [38]. Analytical methods, finite element methods, and meshless methods are often employed to calculate structures with different tensile and compressive moduli. The current analytical solution methods are mainly based on the fiber longitudinal strain criterion presented by Bert [8] and the maximum principal stress criterion presented by Ambartsumyan [39]. Bert’s method, which considers the anisotropy of materials, is primarily used for laminated composites. In contrast, Ambartsumyan’s method, which is based on the maximum principal stress criterion, is mainly applicable to isotropic materials. Building upon these basic methods, several studies have presented analytical solutions for various bimodulus problems [37,40,41], but these have mainly been verified in simple geometric cases such as beams, columns, and plates [42,43]. For complex structures, numerical analysis based on FEM has become an indispensable tool to solve bimodulus problems. Zhang and Wang were pioneers in introducing the finite element analysis method for planar bimodulus structures with circular holes in 1989 [40], noting the poor convergence of traditional iterative schemes when solving nonlinear equations. To enhance convergence, Yang and Wang presented a smoothing technique in 2008 [44], applicable to two-dimensional static bimodular problems and one-dimensional dynamic inverse problems. However, the computational workload and iterative convergence speed are still significantly influenced by the selection of the initial value and parameters of the smoothing function. He et al. in 2009 [41] noted that the shear modulus has a certain influence on the convergence of the algorithm and, based on the Ambartsumyan constitutive model, presented an iterative algorithm for bimodulus materials that considers the impact of the shear modulus. To address the nonlinear problem of bimodulus truss structures, Zhang et al. in 2013 [45] presented a parametric variational principle (PVP) algorithm by introducing the difference between tensile and compressive deformation, extending the algorithm to two-dimensional continua. Pan et al. in 2021 [46] introduced the bimodulus elasticity theory into the mechanical analysis of asphalt pavement for the first time, establishing an efficient finite element numerical calculation method based on bimodulus theory to systematically investigate the mechanical response of typical semi-rigid base asphalt pavement under different tension-compression modulus characteristics. These iterative algorithms based on FEM have demonstrated high convergence speed and calculation accuracy for one-dimensional structures, where the stress distribution is relatively straightforward and the neutral axis can be accurately captured under static loading conditions [18,29,45]. However, for two-dimensional structures, existing models often rely on the assumption of a static neutral layer, neglecting the dynamic shift in the neutral axis under varying load paths and complex stress states. This limitation hinders the accurate simulation of stress redistribution during both static and dynamic loading, particularly in regions where the principal stress directions evolve with deformation [35,36]. Notably, for materials with highly complex or non-uniform internal structures, such as composites or damaged materials, traditional numerical homogenization techniques may become inadequate. In such cases, a combined approach of non-destructive experimental testing and inverse finite element modeling offers a viable alternative pathway to directly extract the macroscopic equivalent elastic properties of the material [47]. Huang et al. in 2018 [48] explored the application of the meshless method (FBM) to bimodulus materials, comparing its computational accuracy and iterative speed with finite element iterative algorithms. Pan et al. in 2020 [34] utilized the meshless method to analyze stress and displacement at the crack tip of bimodulus materials. Yet, due to the limited application of the meshless method in bimodulus problems, further verification is needed to assess its calculation accuracy and convergence speed for complex structures and stress states. Despite these challenges, analysis methods that consider bimodularity have shown higher calculation accuracy and iteration speed for single materials and simple beam and plate structures. However, current models predominantly focus on the behavior in the principal stress directions, often neglecting the complex behavior in non-principal stress directions. Moreover, the accuracy and reliability of existing models when dealing with complex loading paths and multiaxial stress states are yet to be fully established, restricting the broader application of FEM in engineering. Furthermore, the cast iron material model in Abaqus can indeed account for different tensile and compressive yield strengths, flow, and hardening behaviors. However, this model cannot simulate the different elastic moduli in tension and compression, which is the core elastic behavior addressed in this study. Our work specifically targets and resolves this critical limitation.

Modeling in the principal stress directions is straightforward, yet its application is largely confined to idealized scenarios where the principal stress directions align with the material’s principal axes [17]. In contrast, the primary advantage of non-principal stress direction modeling lies in its versatility: by incorporating coordinate transformations, it enables the construction of universal constitutive models applicable to arbitrary stress directions, thereby capturing the true material behavior under complex stress states [15]. Nevertheless, existing bimodulus theoretical models generally lack such comprehensive adaptability and have not systematically integrated non-principal stress directions into their frameworks, limiting their applicability in multiaxial stress or heterogeneous material contexts [8]. Hence, advancing a bimodulus constitutive theory that encompasses both principal and non-principal stress directions is crucial for enhancing the accuracy and engineering relevance of numerical simulations for such materials. This paper extends the existing bimodulus elasticity framework by formulating a constitutive relation applicable to non-principal stress directions, building upon the established theory based on principal stresses. The approach is based on a constitutive formulation that describes material behavior under general stress states, including those not aligned with principal directions.

Within the theoretical framework of bimodular elasticity, constitutive formulations based on stored energy functions have been well established. This approach constructs thermodynamically consistent strain energy potential functions for tensile and compressive mechanical states separately, thereby establishing a complete constitutive relationship. Although such methods possess a rigorous mathematical foundation in theoretical formulation, their finite element implementation requires iterative spectral decomposition of the strain tensor at each material point to determine principal directions. This process is not only computationally prohibitive but also prone to numerical instability. In light of these considerations, the present study adopts an equivalent orthotropic methodology based on transformation theory. This paper maintains the symmetry requirements of the energy-based framework while significantly enhancing computational efficiency, making it particularly suitable for practical numerical simulation of two-dimensional problems.

In addition, this paper has developed a set of advanced finite element models, including the 3-node triangular (TRI3), the 4-node quadrilateral (QUAD4), the 6-node triangular (TRI6), and the 8-node quadrilateral (QUAD8) elements. These models are carefully designed to accurately simulate the complex stress distribution of bimodulus materials in non-principal stress directions. Through rigorous validation against existing simulation software, these models have demonstrated excellent accuracy and reliability. This paper also includes an in-depth analysis of stress–strain curves, providing a new perspective on understanding the behavior of bimodulus materials under different stress states. The rigorous validation of these models not only confirms the effectiveness of the new theoretical framework but also demonstrates its potential in predicting material mechanical behavior.

By combining theoretical innovation and numerical simulation, this paper provides a systematic numerical framework for analyzing bimodulus materials under general stress states. The integration of constitutive modeling with finite element simulation offers a practical approach for studying bimodulus material behavior, which can serve as a basis for future investigations. The developed models support more accurate engineering analysis and design when dealing with materials exhibiting distinct tension–compression properties. As many new materials exhibit significant bimodulus properties, which have an undeniable impact on the mechanical behavior of materials, the models provided in this paper are expected to play an important role in future scientific research and engineering practice.

## 2. Theoretical Background

### 2.1. Principal Stress-Based Bimodulus Model

**Figure 1:**
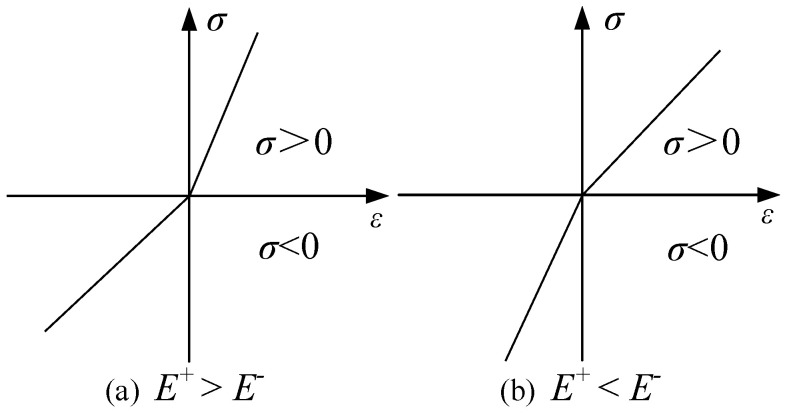
Bilinear stress–strain relationships.

Ambartsumyan’s theory of bimodulus elasticity stipulates that the establishment of the material’s constitutive model is based on the positive or negative signs of the principal stresses as the decisive factor. Therefore, the direction and sign of the principal stresses play a pivotal role. Depending on the signs of the principal stresses, they can be categorized into two types: (1) The first type of point or region, where all principal stresses have the same sign, meaning it is either a fully compressed or fully tensioned area; (2) The second type of point or region, where the sign of one principal stress is opposite to the other two. For the first type of region, the expression of the bimodulus theory of elasticity is the same as that of classical elasticity theory. For the second type of region, Ambartsumyan employs two linear segments to approximate the stress–strain relationship of the bimodulus material; that is, he represents it with piecewise linear segments that have a discontinuous tangent at the origin (see Figure 1). When subjected to tension, the material’s elastic modulus and Poisson’s ratio are represented by $E_{t}$ and $v_{t}$, respectively. Under compression, the material’s elastic modulus and Poisson’s ratio are represented by $E_{c}$ and $v_{c}$, respectively.

According to Ambartsumyan’s theory, for an isotropic elastic body under plane stress conditions, the relationship between principal strains and principal stresses is given by [12]

(1) $$\left\{\begin{array}{c} \varepsilon_{1}\\ \varepsilon_{2} \end{array}\right\}=\left[\begin{array}{cc} s_{11} & s_{12}\\ s_{21} & s_{22} \end{array}\right]\left\{\begin{array}{c} \sigma_{1}\\ \sigma_{2} \end{array}\right\}$$

where $\varepsilon_{1}$ and $\varepsilon_{2}$ represent the principal strains, $\sigma_{1}$ and $\sigma_{2}$ represent the principal stresses, and $s_{ij}\left(i,j=1,2,6\right)$ represent the compliance coefficients in the direction of the principal stresses, which is given by

(2) $$s_{11}=\frac{1}{\frac{E_{t}\;+\;E_{c}}{2}\;+\;\frac{E_{t}\;-\;E_{c}}{2}\mathrm{sgn}\left(\sigma_{1}\right)}$$

(3) $$s_{22}=\frac{1}{\frac{E_{t}\;+\;E_{c}}{2}\;+\;\frac{E_{t}\;-\;E_{c}}{2}\mathrm{sgn}\left(\sigma_{2}\right)}$$

(4) $$s_{12}=-\frac{\nu_{t}}{E_{t}}=-\frac{\nu_{c}}{E_{c}}$$

in which $\mathrm{sgn}\left(\sigma \right)$ represents the sign function, which takes the value of 1 when σ>0 (tension) and the value of −1 when σ<0 (compression). The Young’s modulus exhibits discontinuity at σ=0, and it is commonly assumed that the Young’s modulus at σ=0 is equal to the tensile modulus, where it is assumed that $\mathrm{sgn}\left(0\right)=1$ [11].

In Equation (4), the assumption of $E_{t}\nu_{c}=E_{c}\nu_{t}$ is employed [8]. This assumption is made to ensure the symmetry of the compliance and stiffness matrices, which in turn guarantees the positive definiteness of the strain energy. As a result, the structure exhibits a well-defined mechanical behavior, where an increase in deformation leads to an increase in stored energy. It is emphasized that the present model is developed under the plane stress assumption, and all verifications are conducted within this context. The thickness parameter is incorporated as a constant in the plane stress formulation, without reference to plane strain conditions.

### 2.2. The Axis Transformation Formula

Ambartsumyan’s bilinear stress–strain relationship is established along the principal stress directions. To determine the stress–strain relationship in directions other than the principal stresses, one must utilize the transformation formulas that relate the principal stresses and strains to the off-principal directions.

**Figure 2:**
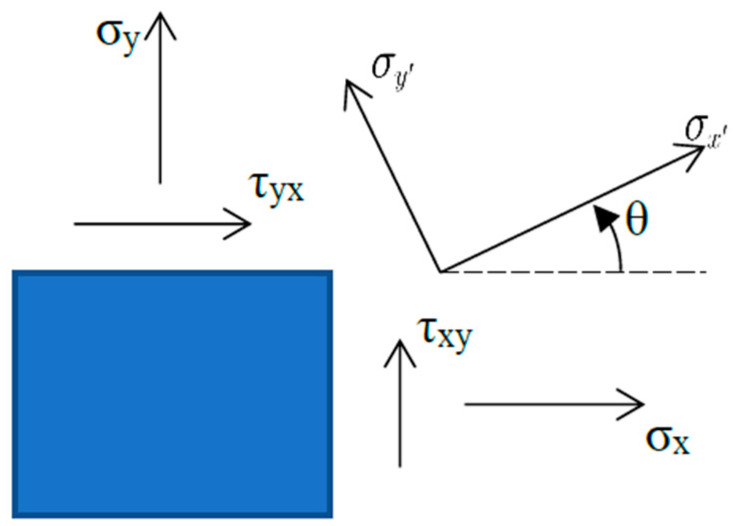
Principal stress directions.

Considering the microelement as shown in Figure 2, the stress components are represented as $\sigma_{x},\sigma_{y},\tau_{xy}$ in the xoy coordinate system. Assuming that the coordinate system xoy is rotated by an angle θ(the angle counter-clockwise from the x-axis to the first principal stress is defined as positive) to a new coordinate system *x′oy′*, according to the stress transformation formula, the stress components in the *x′oy′* coordinate system are given by:

(5) $$\begin{array}{cc} \sigma_{x^{\prime }} & =\sigma_{x}\cos^{2}\;\theta +\sigma_{y}\sin^{2}\;\theta +\tau_{xy}\sin\;2\theta \\ \sigma_{y^{\prime }} & =\sigma_{x}\sin^{2}\;\theta +\sigma_{y}\cos^{2}\;\theta -\tau_{xy}\sin\;2\theta \\ \tau_{x^{\prime }y^{\prime }} & =\left(\sigma_{y}-\sigma_{x}\right)\sin\;\theta \cos\;\theta +\tau_{xy}\cos\;2\theta \end{array}$$

or

(6) $$\left\{\begin{array}{c} \sigma_{x^{\prime }}\\ \sigma_{y^{\prime }}\\ \tau_{x^{\prime }y^{\prime }} \end{array}\right\}=T\left(\theta \right)\sigma$$

where $\sigma ={\left\{\sigma_{x},\sigma_{y},\tau_{xy}\right\}}^{T}$, and

(7) $$T\left(\theta \right)=\left[\begin{array}{ccc} \cos^{2}\;\theta & \sin^{2}\;\theta & \sin\;2\theta \\ \sin^{2}\;\theta & \cos^{2}\;\theta & -\sin\;2\theta \\ -\sin\;\theta \cos\;\theta & \sin\;\theta \cos\;\theta & \cos\;2\theta \end{array}\right]$$

If *x′oy′* is the principal stress direction, then the shear stress $\tau_{x^{\prime }y^{\prime }}$ in that direction is zero. This is because principal stresses act on planes where the shear stress is absent. Mathematically, this can be expressed as $\tau_{x^{\prime }y^{\prime }}=\tau_{12}=0,\;\sigma_{x^{\prime }}=\sigma_{1},\;\sigma_{y^{\prime }}=\sigma_{2}$. Combining Equation (5), the principal stress direction angles can be determined as

(8) $$\theta =\frac{1}{2}\arctan\;\frac{2\tau_{xy}}{\sigma_{x}-\sigma_{y}}$$

This is derived from the perspective of microelement equilibrium; hence, Equation (8) represents the principal stress directions, which are related only to the state of stress and are independent of material properties. It also holds true for materials with different moduli under tension and compression. At this point, Equation (6) is rewritten as

(9) $$\bar{\sigma }=T\left(\theta \right)\sigma$$

where $\bar{\sigma }={\left\{\sigma_{1},\sigma_{2},\tau_{12}\right\}}^{T}$.

Similarly, using the geometric relationship of microelement deformation, the relationship between the strain components in the xoy coordinate system and the strain components in the direction of the principal stress can be obtained as

(10) $$\varepsilon ={\left[T\left(\theta \right)\right]}^{T}\bar{\varepsilon }$$

where $\bar{\varepsilon }={\left\{\varepsilon_{1},\varepsilon_{2},\gamma_{12}\right\}}^{T},\;\varepsilon ={\left\{\varepsilon_{x},\varepsilon_{y},\gamma_{xy}\right\}}^{T}$, ${\left[T\left(\theta \right)\right]}^{T}$ is the transpose of $T\left(\theta \right)$. Due to Equation (10) representing the relationship between the strain components in the xoy coordinate system and the strain components in the direction of the principal stress, which is derived from the geometric relationship of microelement deformation, it is also independent of material properties and holds true for materials with different moduli under tension and compression.

### 2.3. General Stress–Strain Relationship in Non-Principal Stress Directions

For the sake of derivation convenience, even though the shear stress in the principal stress direction is zero, the compliance coefficients related to shear stress are still included in Equation (1). At the same time, it is still assumed that normal stress in the principal stress direction will not produce shear strain. At this point, Equation (1) is rewritten as

(11) $$\bar{\varepsilon }=c\bar{\sigma }$$

where

(12) $$\bar{c}=\left[\begin{array}{ccc} s_{11} & s_{12} & 0\\ s_{21} & s_{22} & 0\\ 0 & 0 & s_{66} \end{array}\right]$$

Substituting (9) into (11) and then substituting the result into (10), one obtains

(13) $$\varepsilon =c\left(\sigma \right)\sigma$$

where $c\left(\sigma \right)$ represents the compliance matrix in the direction of xoy, and

(14) $$c\left(\sigma \right)={\left[T\left(\theta \right)\right]}^{T}\bar{c}T\left(\theta \right)=\left[\begin{array}{ccc} s_{11}^{xy} & s_{12}^{xy} & s_{16}^{xy}\\ s_{12}^{xy} & s_{22}^{xy} & s_{16}^{xy}\\ s_{16}^{xy} & s_{16}^{xy} & s_{66}^{xy} \end{array}\right]$$

Substituting Equation (7) into (14) yields the individual components of the compliance matrix in the xoy direction as

(15) $$s_{11}^{xy}=s_{22}\sin^{2}\;\left(\theta \right)+s_{11}\cos^{2}\;\left(\theta \right)\;\;s_{22}^{xy}=s_{11}\sin^{2}\;\left(\theta \right)+s_{22}\cos^{2}\;\left(\theta \right)\;\;s_{66}^{xy}=s_{11}-2s_{12}+s_{22}\;\;s_{12}^{xy}=s_{12}\;\;s_{16}^{xy}=\left(s_{11}-s_{22}\right)\sin\;\left(\theta \right)\cos\;\left(\theta \right)$$

Through rigorous coordinate transformation, the consistency of the compliance matrix has been verified: the principal stress coordinate system compliance matrix $\bar{c}$ defined by Equation (16) is transformed into the global coordinate system compliance matrix c(σ) via a congruence transformation using the transformation matrix T(θ) and its transpose, i.e., $c(\sigma )={\left[T(\theta )\right]}^{T}\bar{c}T(\theta )$, ultimately yielding the global coordinate system compliance matrix c(σ) in Equation (17). In this process, each component ${s_{ij}}^{xy}$ in Equation (18) is precisely expressed through trigonometric combinations of $s_{11}$, $s_{12}$, $s_{22}$ and $s_{66}$ from Equation (19). Simultaneously, the ordering of the strain–stress vectors is strictly transformed from ${\left\{\left.\varepsilon_{1},\varepsilon_{2},\gamma_{12}\right\}\right.}^{T}$ and ${\left\{\left.\sigma_{1},\sigma_{2},\tau_{12}\right\}\right.}^{T}$ in the principal stress directions to ${\left\{\left.\varepsilon_{x},\varepsilon_{y},\gamma_{xy}\right\}\right.}^{T}$ and ${\left\{\left.\sigma_{x},\sigma_{y},\tau_{xy}\right\}\right.}^{T}$ in the global coordinate directions.

### 2.4. Finite Element Interpolation Functions

This paper aims to construct triangular elements with three and six nodes and rectangular elements with four and eight nodes, suitable for bimodulus materials. According to the relevant theories of the finite element method, interpolation functions for different types of elements are first constructed. For an element with N nodes, the nodes are numbered *i*, *j*, …, *N*, and the coordinates of the nodes are represented as $\left(x_{1},\;y_{1}\right),\;\left(x_{2},\;y_{2}\right),\;\ldots ,\;\left(x_{N},\;y_{N}\right)$. The nodal displacement array is denoted as

(16) $$\delta^{e}={\left\{u_{1},v_{1},u_{2},v_{2},\ldots ,u_{N},v_{N}\right\}}^{T}$$

in which $u_{i}$ and $v_{i}$ represent the displacement of node *i*.

Firstly, a local coordinate system ξOη is introduced, mapping the element from the global coordinate system to the local one. The mapping function is expressed as

(17) $$x=f\left(\xi ,\eta \right),\;y=g\left(\xi \xi ,\eta \right)$$

Utilizing nodal displacements to interpolate the displacement field within the element, the form of the interpolation function is

(18) $$u=N\left(\xi ,\eta \right)\delta^{e}$$

in which $u={\left\{u,v\right\}}^{T}$ represents the displacement field, and N represents the matrix of shape functions for different elements in the local coordinate system.

#### 2.4.1. Triangular Element with Three Nodes

**Figure 3:**
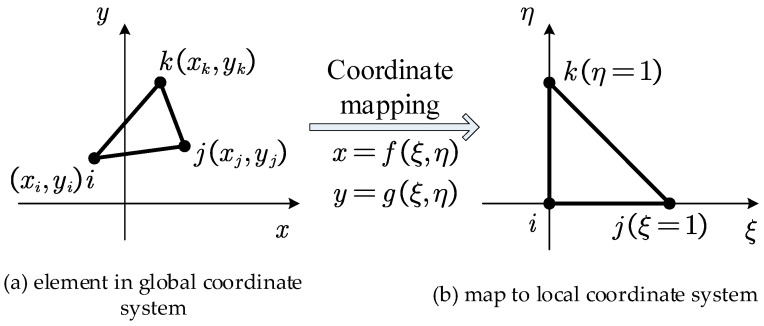
Coordinate mapping of triangular element with three nodes, adapted from [49].

For the triangular element with three nodes, as shown in Figure 3a, local coordinates ξ and η that satisfy Equation (19) are introduced to map the element onto the local coordinate system shown in Figure 3b.

(19) $$\begin{array}{c} x=f\left(\xi ,\eta \right)=\xi \left(x_{j}-x_{i}\right)+\eta \left(x_{k}-x_{i}\right)+x_{i}\\ y=g\left(\xi ,\eta \right)=\xi \left(y_{j}-y_{i}\right)+\eta \left(y_{k}-y_{i}\right)+y_{i} \end{array}$$

The nodal displacement array for the element is denoted as

(20) $$\delta^{e}={\left\{u_{i},v_{i},u_{j},v_{j},u_{k},v_{k}\right\}}^{T}$$

The interpolation function is given by

(21) $$u=N\delta^{e}$$

in which N is the matrix of shape functions, and

(22) $$N=\left[N_{i}I,N_{j}I,N_{k}I\right]$$

and

(23) $$I=\left[\begin{array}{cc} 1 & 0\\ 0 & 1 \end{array}\right]$$

(24) $$N_{i}=1-\xi ,\;N_{j}=\xi ,\;N_{k}=\eta$$

#### 2.4.2. Rectangular Element with Four Nodes

**Figure 4:**
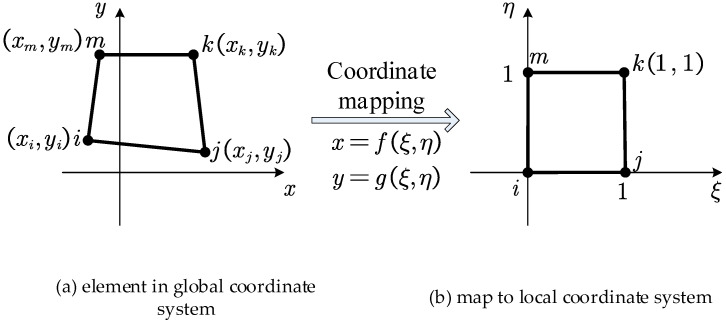
Rectangular element with four nodes.

For the rectangular element with four nodes, as shown in Figure 4a, local coordinates ξ and η that satisfy Equation (25) are introduced to map the element onto the local coordinate system shown in Figure 4b.

(25) $$x=f\left(\xi ,\eta \right)=\xi \eta \left(x_{i}-x_{j}+x_{k}-x_{m}\right)+\xi \left(x_{j}-x_{i}\right)+\eta \left(x_{m}-x_{i}\right)+x_{i}\;\;y=g\left(\xi ,\eta \right)=\xi \eta \left(y_{i}-y_{j}+y_{k}-y_{m}\right)+\xi \left(y_{j}-y_{i}\right)+\eta \left(y_{m}-y_{i}\right)+y_{i}$$

The nodal displacement array for the element is denoted as

(26) $$\delta^{e}={\left\{u_{i},v_{i},u_{j},v_{j},u_{k},v_{k},u_{m},v_{m}\right\}}^{T}$$

The interpolation function is given by

(27) $$u=N\delta^{e}$$

where

(28) $$N=\left[N_{i}I,N_{j}I,N_{k}I,N_{m}I\right]$$

(29) $$N_{i}=1-\xi -\eta +\xi \eta \;\;N_{j}=\xi -\xi \eta \;\;N_{k}=\xi \eta \;\;N_{m}=\eta -\xi \eta$$

#### 2.4.3. Triangular Element with Six Nodes

**Figure 5:**
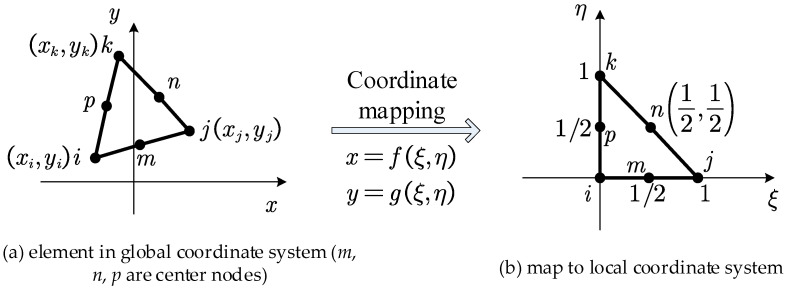
Coordinate mapping of triangular element with six nodes.

For the triangular element with six nodes, as shown in Figure 5a, local coordinates ξ and η that satisfy Equation (30) are introduced to map the element onto the local coordinate system shown in Figure 5b.

(30) $$\begin{array}{cc} x & =\;\xi^{2}\left(2x_{i}+2x_{j}-4x_{m}\right)+\xi \left(-3x_{i}-x_{j}+4x_{m}\right)\\ & +\;\eta^{2}\left(2x_{i}+2x_{k}-4x_{p}\right)+\eta \left(-3x_{i}-x_{k}+4x_{p}\right)\\ & +\;\eta \xi \left(4x_{i}-4x_{m}+4x_{n}-4x_{p}\right)+x_{i}\\ y & =\;\xi^{2}\left(2y_{i}+2y_{j}-4y_{m}\right)+\xi \left(-3y_{i}-y_{j}+4y_{m}\right)\\ & +\;\eta^{2}\left(2y_{i}+2y_{k}-4y_{p}\right)+\eta \left(-3y_{i}-y_{k}+4y_{p}\right)\\ & +\;\eta \xi \left(4y_{i}-4y_{m}+4y_{n}-4y_{p}\right)+y_{i} \end{array}$$

The nodal displacement array for the element is denoted as

(31) $$\delta^{e}={\left\{u_{i},v_{i},u_{j},v_{j},u_{k},v_{k},u_{m},v_{m},u_{n},v_{n},u_{p},v_{p}\right\}}^{T}$$

The interpolation function is given by

(32) $$u=N\delta^{e}$$

where

(33) $$N=\left[N_{i}I,N_{j}I,N_{k}I,N_{m}I,N_{n}I,N_{p}I\right]$$

(34) $$\begin{array}{cc} N_{i} & =\;2\eta^{2}+4\eta \xi -3\eta +2\xi^{2}-3\xi +1\\ N_{j} & =\;2\xi^{2}-\xi \\ N_{k} & =\;2\eta^{2}-\eta \\ N_{m} & =\;-4\eta \xi -4\xi^{2}+4\xi \\ N_{n} & =\;4\eta \xi \\ N_{p} & =\;-4\eta^{2}-4\eta \xi +4\eta \end{array}$$

#### 2.4.4. Rectangular Element with Eight Nodes

**Figure 6:**
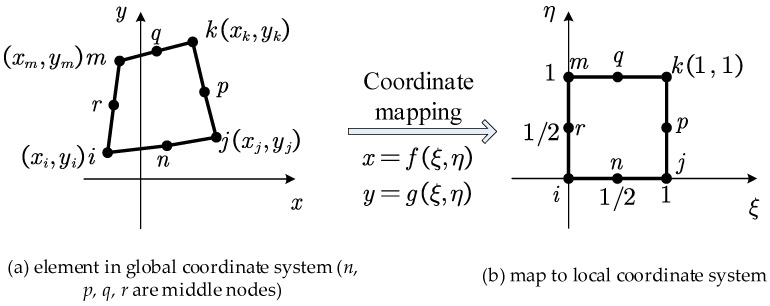
Coordinate mapping of rectangular element with eight nodes.

For the rectangular element with eight nodes, as shown in Figure 6a, local coordinates ξ and η that satisfy Equation (35) are introduced to map the element onto the local coordinate system shown in Figure 6b.

(35) $$\begin{array}{cc} x & =\;\eta \xi \left(5x_{i}-x_{j}-3x_{k}-x_{m}-4x_{n}+4x_{p}+4x_{q}-4x_{r}\right)\\ & +\;\eta \xi^{2}\left(-2x_{i}-2x_{j}+2x_{k}+2x_{m}+4x_{n}-4x_{q}\right)\\ & +\;\eta^{2}\xi \left(-2x_{i}+2x_{j}+2x_{k}-2x_{m}-4x_{p}+4x_{r}\right)\\ & +\;\xi^{2}\left(2x_{i}+2x_{j}-4x_{n}\right)+\xi \left(-3x_{i}-x_{j}+4x_{n}\right)\\ & +\;\eta^{2}\left(2x_{i}+2x_{m}-4x_{r}\right)+\eta \left(-3x_{i}-x_{m}+4x_{r}\right)+x_{i}\\ y & =\;\eta \xi \left(5y_{i}-y_{j}-3y_{k}-y_{m}-4y_{n}+4y_{p}+4y_{q}-4y_{r}\right)\\ & +\;\eta \xi^{2}\left(-2y_{i}-2y_{j}+2y_{k}+2y_{m}+4y_{n}-4y_{q}\right)\\ & +\;\eta^{2}\xi \left(-2y_{i}+2y_{j}+2y_{k}-2y_{m}-4y_{p}+4y_{r}\right)\\ & +\;\xi^{2}\left(2y_{i}+2y_{j}-4y_{n}\right)+\xi \left(-3y_{i}-y_{j}+4y_{n}\right)\\ & +\;\eta^{2}\left(2y_{i}+2y_{m}-4y_{r}\right)+\eta \left(-3y_{i}-y_{m}+4y_{r}\right)+y_{i} \end{array}$$

The nodal displacement array for the element is denoted as

(36) $$\delta^{e}={\left\{u_{i},v_{i},u_{j},v_{j},u_{k},v_{k},u_{m},v_{m},u_{n},v_{n},u_{p},v_{p},u_{q},v_{q},u_{r},v_{r}\right\}}^{T}$$

The interpolation function is given by

(37) $$u=N\delta^{e}$$

where

(38) $$N=\left[N_{i}I,N_{j}I,N_{k}I,N_{m}I,N_{n}I,N_{p}I,N_{q}I,N_{r}I\right]$$

(39) $$\begin{array}{cc} N_{i} & =-2\eta^{2}\xi +2\eta^{2}-2\eta \xi^{2}+5\eta \xi -3\eta +2\xi^{2}-3\xi +1\\ N_{j} & =2\eta^{2}\xi -2\eta \xi^{2}-\eta \xi +2\xi^{2}-\xi \\ N_{k} & =2\eta^{2}\xi +2\eta \xi^{2}-3\eta \xi \\ N_{m} & =-2\eta^{2}\xi +2\eta^{2}+2\eta \xi^{2}-\eta \xi -\eta \\ N_{n} & =4\eta \xi^{2}-4\eta \xi -4\xi^{2}+4\xi \\ N_{p} & =4\eta \xi -4\eta^{2}\xi \\ N_{q} & =4\eta \xi -4\eta \xi^{2}\\ N_{r} & =4\eta^{2}\xi -4\eta^{2}-4\eta \xi +4\eta \end{array}$$

### 2.5. Element Equations

According to the theory of elasticity, the geometric equation is

(40) ε=Lu

In which L represents the differential operator, with the expression being

(41) $$L=\left[\begin{array}{cc} \frac{\partial }{\partial x} & 0\\ 0 & \frac{\partial }{\partial y}\\ \frac{\partial }{\partial y} & \frac{\partial }{\partial x} \end{array}\right]$$

Substituting Equation (18) into Equation (40) yields the strain field as

(42) $$\varepsilon =LN\delta^{e}=B\delta^{e}$$

in which B represents the geometric matrix, and

(43) B=LN

It should be noted that the differential operator L is related to the derivatives with respect to the absolute coordinates x and y, while the shape function matrix N is a function of the relative coordinates ξ and η. Therefore, the chain rule of differentiation must be used to rewrite L in terms of ξ and η. From the parametric variation relationship (17), it is straightforward to obtain the partial derivatives of the global coordinates with respect to the relative coordinates. According to the rules of differentiation, the following is obtained

(44) $$\frac{\partial \left(\cdot \right)}{\partial \xi }=\frac{\partial x}{\partial \xi }\frac{\partial \left(\cdot \right)}{\partial x}+\frac{\partial y}{\partial \xi }\frac{\partial \left(\cdot \right)}{\partial y}$$

(45) $$\frac{\partial \left(\cdot \right)}{\partial \eta }=\frac{\partial x}{\partial \eta }\frac{\partial \left(\cdot \right)}{\partial x}+\frac{\partial y}{\partial \eta }\frac{\partial \left(\cdot \right)}{\partial y}$$

The last two equations can be rewritten in matrix form as

(46) $$\left\{\begin{array}{c} \frac{\partial \left(\cdot \right)}{\partial \xi }\\ \frac{\partial \left(\cdot \right)}{\partial \eta } \end{array}\right\}=J\left\{\begin{array}{c} \frac{\partial \left(\cdot \right)}{\partial x}\\ \frac{\partial \left(\cdot \right)}{\partial y} \end{array}\right\}$$

in which J represents the Jacobian matrix, and

(47) $$J=\left[\begin{array}{cc} \frac{\partial x}{\partial \xi } & \frac{\partial y}{\partial \xi }\\ \frac{\partial x}{\partial \eta } & \frac{\partial y}{\partial \eta } \end{array}\right]\left\{\begin{array}{c} \frac{\partial \left(\cdot \right)}{\partial x}\\ \frac{\partial \left(\cdot \right)}{\partial y} \end{array}\right\}$$

This facilitates the easy establishment of the relationship between the derivatives of the shape functions with respect to the absolute coordinates and those with respect to the relative coordinates.

(48) $$\left\{\begin{array}{c} \frac{\partial \left(\cdot \right)}{\partial x}\\ \frac{\partial \left(\cdot \right)}{\partial y} \end{array}\right\}=J^{-1}\left\{\begin{array}{c} \frac{\partial \left(\cdot \right)}{\partial \xi }\\ \frac{\partial \left(\cdot \right)}{\partial \eta } \end{array}\right\}$$

Substituting Equation (42) into (13), one obtains that

(49) $$c\left(\sigma \right)\sigma =B\delta^{e}$$

or

(50) $$\sigma ={\left[c\left(\sigma \right)\right]}^{-1}B\delta^{e}$$

Substituting the stress components from (50) into (8) yields the expression for the principal stress direction angles in terms of nodal displacements. For different elements, the expression relating the principal stress direction angles to nodal displacements varies and will be presented in Section 2.6.

For bimodulus materials, the principle of strain energy still applies. For planar elements, the principle of strain energy is expressed as

(51) $$\int_{\Omega^{e}}{\left(\delta \varepsilon \right)}^{T}\sigma d\Omega -{\left(\delta \delta^{e}\right)}^{T}f^{e}=0$$

in which $f^{e}$ represents the nodal force array, δ represents the variational symbol, and $\Omega^{e}$ represents the volume of the element. Substituting (42) and (50) into (51) yields

(52) $${\left(\delta \delta^{e}\right)}^{T}\left(K^{e}\delta^{e}-f^{e}\right)=0$$

where

(53) $$K^{e}=t\int_{A^{e}}B^{T}{\left[c\left(\sigma \right)\right]}^{-1}BdA$$

The element stiffness matrix in Equation (53) involves an integral over the element in absolute coordinates. It is necessary to rewrite this integral in terms of local coordinates using the elemental relationships to facilitate solution. The transformation relationship for the differential area element dA is given by

(54) $$dA=d\xi d\eta \sqrt{{\left(\frac{\partial x}{\partial \xi }\frac{\partial y}{\partial \eta }-\frac{\partial x}{\partial \eta }\frac{\partial y}{\partial \xi }\right)}^{2}}$$

### 2.6. Principal Stress Direction Angles of the Element

Different elements possess distinct simulation capabilities for the direction of principal stresses. The principal stress direction angles, derived by substituting the stress components from Equation (50) into Equation (8), can be formulated as expressions dependent on the nodal displacements of the element. Here, the expressions for the principal stress direction angles of the four models presented in this paper are presented.

#### 2.6.1. Triangular Element with Three Nodes

According to Equation (48), in conjunction with Equation (41) and the shape function matrix (22) of the triangular element with three nodes, the specific form of the geometric matrix B is obtained as

(55) $$B=\left(\begin{array}{cccccc} b_{11} & 0 & b_{13} & 0 & b_{15} & 0\\ 0 & b_{22} & 0 & b_{24} & 0 & b_{26}\\ b_{22} & b_{11} & b_{24} & b_{13} & b_{26} & b_{15} \end{array}\right)$$

where

(56) $$b_{11}=\frac{y_{j}-y_{k}}{\det\left(J\right)},b_{13}=\frac{y_{k}-y_{i}}{\det\left(J\right)},b_{15}=\frac{y_{i}-y_{j}}{\det\left(J\right)}\;\;b_{22}=\frac{x_{k}-x_{j}}{\det\left(J\right)},b_{24}=\frac{x_{i}-x_{k}}{\det\left(J\right)},b_{26}=\frac{x_{j}-x_{i}}{\det\left(J\right)}\;\;\det\left(J\right)=-x_{j}y_{i}+x_{i}y_{j}+y_{i}x_{k}-x_{i}y_{k}-y_{j}x_{k}+x_{j}y_{k}$$

Substituting Equation (55) into Equation (50) yields the stress components in the xoy coordinate system. Combining this with Equation (8) allows for the determination of the relationship between the principal stress directions within the element and the nodal displacements of the element as follows:

(57) $$\theta =\frac{1}{2}\arctan\frac{u_{i}b_{22}+u_{j}b_{24}+u_{k}b_{26}+v_{i}b_{11}+v_{j}b_{13}+v_{k}b_{15}}{u_{i}b_{11}+u_{j}b_{13}+u_{k}b_{15}-v_{i}b_{22}-v_{j}b_{24}-v_{k}b_{26}}$$

It can be observed that for an element, the principal stress direction angles can be determined by the nodal displacements, thereby obtaining the principal stress directions within the element, as well as the signs of the principal stresses to ascertain the elastic compliance coefficients. Ultimately, this leads to the acquisition of the element’s stiffness matrix.

Since the nodal displacements are initially unknown, an iterative solution is required. An initial displacement field is assumed, and then the principal stress direction angles within the element are determined. Combining this with Equation (50) and Equation (9), the signs of the principal stresses are established. Based on the signs of the principal stresses, the element’s elastic compliance matrix is updated using Equations (2)–(4), the overall stiffness matrix is assembled, and a new displacement field is obtained. This process is iterated until the displacements converge.

#### 2.6.2. Quadrilateral Element with Four Nodes

According to Equation (48), in conjunction with Equation (41) and the shape function matrix (28) of the quadrilateral element with four nodes, the form of the geometric matrix B is determined as

(58) $$B=\left(\begin{array}{cccccccc} b_{11} & 0 & b_{13} & 0 & b_{15} & 0 & b_{17} & 0\\ 0 & b_{22} & 0 & b_{24} & 0 & b_{26} & 0 & b_{28}\\ b_{22} & b_{11} & b_{24} & b_{13} & b_{26} & b_{15} & b_{28} & b_{17} \end{array}\right)$$

where

(59) $$\begin{array}{cc} b_{11} & =\frac{\left(1-\eta \right)y_{j}+y_{k}(\eta -\xi )+(\xi -1)y_{m}}{\det\left(J\right)},\;b_{13}=\frac{\left(\eta -1)y_{i}+\xi y_{k}-y_{m}(\eta +\xi -1\right)}{\det\left(J\right)}\\ b_{15} & =\frac{y_{i}(\xi -\eta )-\xi y_{j}+\eta y_{m}}{\det\left(J\right)},b_{17}=\frac{\left(1-\xi \right)y_{i}+y_{j}(\eta +\xi -1)-\eta y_{k}}{\det\left(J\right)}\\ b_{22} & =\frac{(\eta -1)x_{j}+x_{k}(\xi -\eta )+\left(1-\xi \right)x_{m}}{\det\left(J\right)},b_{24}=\frac{\left(1-\eta \right)x_{i}-\xi x_{k}+x_{m}(\eta +\xi -1)}{\det\left(J\right)}\\ b_{26} & =\frac{\eta x_{i}-\xi x_{i}+\xi x_{j}-\eta x_{m}}{\det\left(J\right)},b_{28}=\frac{(\xi -1)x_{i}-x_{j}(\eta +\xi -1)+\eta x_{k}}{\det\left(J\right)}\\ \det\left(J\right) & =\left(1-\eta \right)x_{i}y_{j}+\left(\eta -\xi \right)x_{i}y_{k}+\left(\xi -1\right)x_{i}y_{m}+\left(\eta -1\right)x_{j}y_{i}\\ & +\left(1-\eta -\xi \right)x_{j}y_{m}+\left(\xi -\eta \right)x_{k}y_{i}+\left(1-\xi \right)x_{m}y_{i}+\left(\eta +\xi \right)x_{m}y_{j}\\ & +\;\xi x_{j}y_{k}-\xi x_{k}y_{j}+\eta x_{k}y_{m}-x_{m}y_{j}-\eta x_{m}y_{k} \end{array}$$

Substituting Equation (58) into Equation (50) yields the stress components in the xoy coordinate system. Combining this with Equation (8) allows for the determination of the relationship between the principal stress directions within the element and the nodal displacements of the element as follows:

(60) $$\theta =\frac{1}{2}\arctan\frac{u_{i}b_{22}+u_{j}b_{24}+u_{k}b_{26}+u_{m}b_{28}+v_{i}b_{11}+v_{j}b_{13}+v_{k}b_{15}+v_{m}b_{17}}{u_{i}b_{11}+u_{j}b_{13}+u_{k}b_{15}+u_{m}b_{17}-v_{i}b_{22}-v_{j}b_{24}-v_{k}b_{26}-v_{m}b_{28}}$$

#### 2.6.3. Triangular Element with Six Nodes

According to Equation (48), in conjunction with Equation (41) and the shape function matrix (33) of the triangular element with six nodes, the form of the geometric matrix B is determined as

(61) $$B=\left(\begin{array}{cccccccccccc} b_{11} & 0 & b_{13} & 0 & b_{15} & 0 & b_{17} & 0 & b_{19} & 0 & b_{111} & 0\\ 0 & b_{22} & 0 & b_{24} & 0 & b_{26} & 0 & b_{28} & 0 & b_{210} & 0 & b_{212}\\ b_{22} & b_{11} & b_{24} & b_{13} & b_{26} & b_{15} & b_{28} & b_{17} & b_{210} & b_{19} & b_{212} & b_{111} \end{array}\right)$$

where

(62) $$\begin{array}{c} b_{11}=\frac{\left(4\eta +4\xi -3\right)}{\det\left(J\right)}\left[\begin{array}{c} \left(1-4\xi \right)y_{j}+y_{k}(4\eta -1)+4y_{n}(\xi -\eta )\\ +4y_{m}(\eta +\xi -1)-4y_{p}(\eta +\xi -1) \end{array}\right]\\ b_{13}=\frac{\left(4\xi -1\right)}{\det\left(J\right)}\left[\begin{array}{c} y_{k}(4\eta -1)+4(y_{n}-y_{m})\xi \\ -4y_{p}(2\eta +\xi -1)+y_{i}(4\eta +4\xi -3) \end{array}\right]\\ b_{15}=\frac{\left(4\eta -1\right)}{\det\left(J\right)}\left[\begin{array}{c} \left(1-4\xi \right)y_{j}+4y_{m}\left(\eta +2\xi -1\right)-4\eta y_{n}\\ +4y_{p}\eta +y_{i}(-4\eta -4\xi +3) \end{array}\right]\\ b_{17}=-\frac{4}{\det\left(J\right)}\left(\begin{array}{l} 4y_{p}\left(3\xi -2\eta^{2}+3\eta -4\xi \eta -2\xi^{2}-1\right)\\ +y_{i}\left[4\eta^{2}+(8\xi -7)\eta +4\xi^{2}-7\xi +3\right]\\ +y_{j}\left(\xi -4\xi^{2}\right)+4y_{n}\left(2\xi^{2}-\xi \right)+y_{k}(4\eta -1)(\eta +2\xi -1) \end{array}\right)\\ b_{19}=\frac{4}{\det\left(J\right)}\left(\begin{array}{l} -8y_{p}\eta^{2}+4y_{p}\eta +y_{k}(4\eta -1)\eta -4y_{j}\xi^{2}\\ +8y_{m}\xi^{2}+y_{j}\xi -4y_{m}\xi +y_{i}\left(4\eta^{2}-3\eta +\xi (3-4\xi )\right) \end{array}\right)\\ b_{111}=\frac{4}{\det\left(J\right)}\left(\begin{array}{l} -4y_{k}\eta^{2}-8y_{m}\eta^{2}+8y_{n}\eta^{2}+y_{k}\eta +12y_{m}\eta \\ -4y_{n}\eta -16y_{m}\xi \eta -8y_{m}\xi^{2}-4y_{m}+12y_{m}\xi \\ +y_{j}(2\eta +\xi -1)(4\xi -1)+y_{i}\left(4\eta^{2}+(8\xi -7)\eta +4\xi^{2}-7\xi +3\right) \end{array}\right)\\ b_{22}=\frac{\left(4\eta +4\xi -3\right)}{\det\left(J\right)}\left(\begin{array}{l} -4\eta x_{k}+x_{k}+4x_{n}(\eta -\xi )\\ -4x_{m}(\eta +\xi -1)+4x_{p}(\eta +\xi -1)+x_{j}(4\xi -1) \end{array}\right)\\ b_{24}=\frac{\left(4\xi -1\right)}{\det\left(J\right)}\left(\begin{array}{c} -4\eta x_{k}+x_{k}-4x_{p}+8x_{p}\eta +4x_{m}\xi \\ -4x_{n}\xi +4x_{p}\xi +x_{i}(-4\eta -4\xi +3) \end{array}\right)\\ b_{26}=\frac{\left(4\eta -1\right)}{\det\left(J\right)}\left(\begin{array}{c} 4(x_{n}-x_{p})\eta -4x_{m}(\eta +2\xi -1)\\ +x_{j}(4\xi -1)+x_{i}(4\eta +4\xi -3) \end{array}\right)\\ b_{28}=\frac{4}{\det\left(J\right)}\left(\begin{array}{l} -8x_{p}\eta^{2}+12x_{p}\eta -16x_{p}\xi \eta -4x_{j}\xi^{2}+8x_{n}\xi^{2}\\ -8x_{p}\xi^{2}-4x_{p}+x_{j}\xi -4x_{n}\xi +12x_{p}\xi \\ +x_{k}(4\eta -1)(\eta +2\xi -1)+x_{i}\left(4\eta^{2}+(8\xi -7)\eta +4\xi^{2}-7\xi +3\right) \end{array}\right)\\ b_{210}=-\frac{4}{\det\left(J\right)}\left(\begin{array}{l} -8x_{p}\eta^{2}+4x_{p}\eta +x_{k}(4\eta -1)\eta -4x_{j}\xi^{2}\\ +8x_{m}\xi^{2}+x_{j}\xi -4x_{m}\xi +x_{i}\left(4\eta^{2}-3\eta +\xi (3-4\xi )\right) \end{array}\right)\\ b_{212}=-\frac{4}{\det\left(J\right)}\left(\begin{array}{l} -4x_{k}\eta^{2}-8x_{m}\eta^{2}+8x_{n}\eta^{2}+x_{k}\eta +12x_{m}\eta \\ -4x_{n}\eta -16x_{m}\xi \eta -8x_{m}\xi^{2}-4x_{m}+12x_{m}\xi \\ +x_{j}(2\eta +\xi -1)(4\xi -1)+x_{i}\left(4\eta^{2}+(8\xi -7)\eta +4\xi^{2}-7\xi +3\right) \end{array}\right) \end{array}$$

Substituting Equation (61) into Equation (50) yields the stress components in the xoy coordinate system. By combining this with Equation (8), the relationship between the principal stress directions within the element and the nodal displacements of the element can be determined as follows:

(63) $$\begin{array}{cc} \theta & =\frac{1}{2}\arctan\frac{\left(*\right)}{\left(**\right)}\\ \left(*\right) & =u_{i}b_{22}+u_{j}b_{24}+u_{k}b_{26}+u_{m}b_{28}+u_{n}b_{210}\\ & +\;u_{p}b_{212}+v_{i}b_{11}+v_{j}b_{13}+v_{k}b_{15}+v_{m}b_{17}+v_{n}b_{19}+v_{p}b_{111}\\ \left(**\right) & =u_{i}b_{11}+u_{j}b_{13}+u_{k}b_{15}+u_{m}b_{17}+u_{n}b_{19}\\ & +\;u_{p}b_{111}-v_{i}b_{22}-v_{j}b_{24}-v_{k}b_{26}-v_{m}b_{28}-v_{n}b_{210}-v_{p}b_{212} \end{array}$$

#### 2.6.4. Quadrilateral Element with Eight Nodes

According to Equation (48), in conjunction with Equation (41) and the shape function matrix (38) of the quadrilateral element with eight nodes, the form of the geometric matrix B is determined as

(64) $$B=\left(\begin{array}{cccccccccccccccc} b_{11} & 0 & b_{13} & 0 & b_{15} & 0 & b_{17} & 0 & b_{19} & 0 & b_{111} & 0 & b_{113} & 0 & b_{115} & 0\\ 0 & b_{22} & 0 & b_{24} & 0 & b_{26} & 0 & b_{28} & 0 & b_{210} & 0 & b_{212} & 0 & b_{214} & 0 & b_{216}\\ b_{22} & b_{11} & b_{24} & b_{13} & b_{26} & b_{15} & b_{28} & b_{17} & b_{210} & b_{19} & b_{212} & b_{111} & b_{214} & b_{113} & b_{216} & b_{115} \end{array}\right)$$

Substituting Equation (64) into Equation (50) yields the stress components in the xoy coordinate system. By combining this with Equation (8), the relationship between the principal stress directions within the element and the nodal displacements of the element can be determined as follows:

(65) $$\begin{array}{cc} \theta & =\frac{1}{2}\arctan\frac{\left(*\right)}{\left(**\right)}\\ \left(*\right) & =-u_{i}b_{22}-u_{j}b_{24}-u_{k}b_{26}-u_{m}b_{28}-u_{n}b_{210}-u_{p}b_{212}-u_{q}b_{214}\\ & -\;u_{r}b_{216}-v_{i}b_{11}-v_{j}b_{13}-v_{k}b_{15}-v_{m}b_{17}-v_{n}b_{19}-v_{p}b_{111}-v_{q}b_{113}-v_{r}b_{115}\\ \left(**\right) & =-u_{i}b_{11}-u_{j}b_{13}-u_{k}b_{15}-u_{m}b_{17}-u_{n}b_{19}-u_{p}b_{111}-u_{q}b_{113}-u_{r}b_{115}\\ & +\;v_{i}b_{22}+v_{j}b_{24}+v_{k}b_{26}+v_{m}b_{28}+v_{n}b_{210}+v_{p}b_{212}+v_{q}b_{214}+v_{r}b_{216} \end{array}$$

### 2.7. Overall Equations

A discretized model of a structure consists of many elements. For the discretized global structure, the principle of strain energy is expressed as

(66) $$\begin{array}{c} \sum_{e=1}^{N}\left(\int_{\Omega^{e}}{\left(\delta \varepsilon \right)}^{T}\sigma d\Omega \right)\\ -\sum_{e=1}^{N}\left[\int_{\Omega^{e}}{\left(\delta u\right)}^{T}fd\Omega +\int_{S_{e}}{\left(\delta u\right)}^{T}f_{S}dS+{\left(\delta \delta^{e}\right)}^{T}f_{p}\right]=0 \end{array}$$

in which f represents the body forces within the element, $f_{s}$ represents the surface tractions on the element, $f_{p}$ represents the nodal load vector of the element, and $S^{e}$ represents the displacement boundary of the element.

Substituting (42) and (50) into Equation (66) yields

(67) $${\left(\delta \delta \right)}^{T}\left(K\delta -F\right)=0$$

where

(68) $$K=\sum_{e=1}^{N}K^{e},\delta =\sum_{e=1}^{N}\delta^{e},F=\sum_{e=1}^{N}F^{e}$$

representing the global stiffness matrix, the global nodal displacement array, and the global load array. The global load array is calculated as the sum of the element load arrays, and the expression for the element load array is given by

(69) $$F^{e}=f_{p}+\int_{\Omega^{e}}N^{T}fd\Omega +\int_{S_{e}}N^{T}f_{s}dS$$

Given the arbitrariness of δδ, the global stiffness equation can be obtained as

(70) Kδ=F

## 3. Solution Methods and Model Verification

### 3.1. Solution Methods

Considering the bimodulus characteristics of planar problems, the stiffness matrix is related to the stress state. Specifically, on one hand, the components of the element flexibility matrix are related to the principal stress direction angles (see Equation (15)), and on the other hand, the flexibility coefficients of the principal stress directions are related to the positive or negative signs of the principal stresses (see Equation (2)). Therefore, iterative solutions are required when solving such problems.

Each element is divided into 50 points, and T-scheme integration (stress recovery calculation and θ update adopt the same integration rule) is performed on a 50 × 50 grid. Direct iteration is applied to the subdivided mesh elements. If the computed results do not meet the requirements, the process enters a loop, with no maximum iteration limit set. It is worth noting that in the computed results, although the value of $E_{t}$ or $E_{c}$ may switch, the variation in principal stress within the elements is smooth, thus avoiding data oscillation and ensuring that the convergence path is reproducible without relying on empirical adjustments.

The iteration process involves two levels: element iteration and global iteration. In the global iteration, for a given external load, the overall displacement field is initially assumed to be zero. Through this assumed displacement field, the element iteration is entered to derive the element stiffness matrix, which is related to the magnitude and sign of the principal stresses. After assembling the overall stiffness matrix according to Equation (68), the new displacement field is solved using Equation (70). Then, a new iteration step is carried out using the solved displacement field, and this process continues until the displacement converges $\frac{\mathrm{norm}(U_{n-1}-U_{n})}{\mathrm{norm}(U_{n})}\;<\;1\;\times \;10^{-6}$ (Corresponding to Algorithm 1).

For the element iteration, first consider that the element is in a fully tensioned state, with the principal stress direction angle being 0. Using nodal displacements, the principal stress direction angles of each element are calculated through Equations (57), (60), (63) or (65). The sign of the principal stresses is determined using Equations (50) and (11), and the element’s flexibility coefficient matrix is updated. Then, a new element iteration is performed using the derived principal stress state until the principal stress direction within the element converges. After the element iteration converges $\mathrm{norm}(\sigma_{1_{n-1}}-\sigma_{1_{n}})=0$ and $\mathrm{norm}(\sigma_{2_{n-1}}-\sigma_{2_{n}})=0$. (Corresponding to Algorithm 2) Here, a norm of zero indicates that the tension–compression state within all elements has stabilized; the element stiffness matrix is obtained according to Equations (14), (15), (43), and (53), and the process returns to the global iteration.

**Figure 7:**
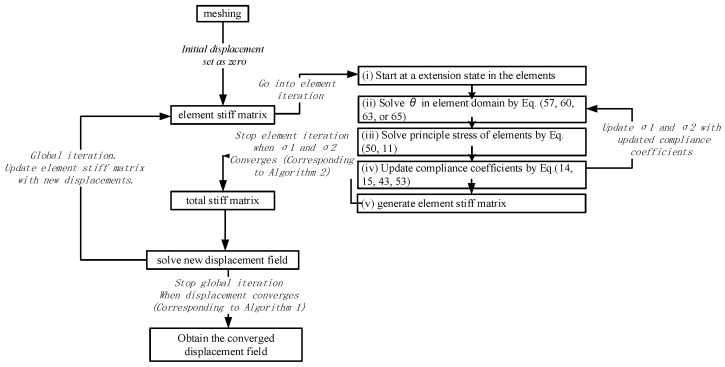
Iterative solution flowchart.

The flowchart for the iterative solution is shown in Figure 7.
**Algorithm 1** Code block to determine if displacement breaks out of the loop1 if norm(totalDisplaceOld − totalDisplace)/norm(totalDisplace) < 1e-62 break;3 end

**Algorithm 2** Code block to determine if σ1 and σ2 break out of the loop1 if norm(sigma1_new-sigma1) == 0 && norm(sigma2_new-sigma2) == 02 break;3 end

### 3.2. Model Verification

**Figure 8:**
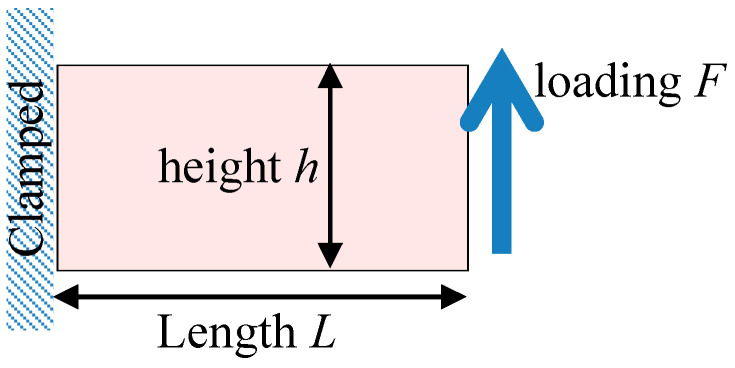
Geometry of the beam.

Consider a plate with a length L of 0.2 m, a height h of 0.1 m, and a thickness b of 0.2 m, with the left end fixed and the right end subjected to an in-plane load. The geometric model is shown in Figure 8.

**Figure 9:**
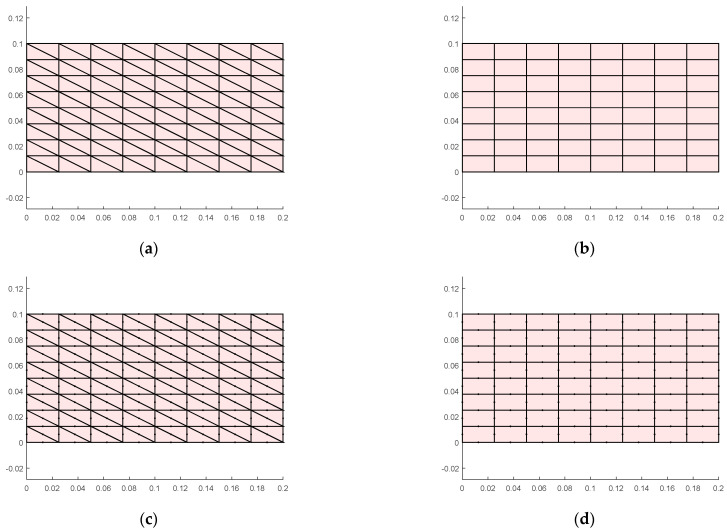
Meshing ($n_{div}=8$): (**a**) triangle element with 3 nodes, (**b**) quadrilateral element with 4 nodes, (**c**) triangle element with 6 nodes, (**d**) quadrilateral element with 8 nodes.

Calculations are performed using the four-element models presented in this paper. The number of mesh lines along each edge is denoted as $n_{div}$, and the meshed structure is shown in Figure 9.

For convenience, in the following analysis, the three-node triangular element is abbreviated as TRI3, the four-node quadrilateral element as QUAD4, the six-node triangular element as TRI6, and the eight-node quadrilateral element as QUAD8.

#### 3.2.1. Verification by Degeneration to Tension–Compression Isotropic Modulus Model

**Figure 10:**
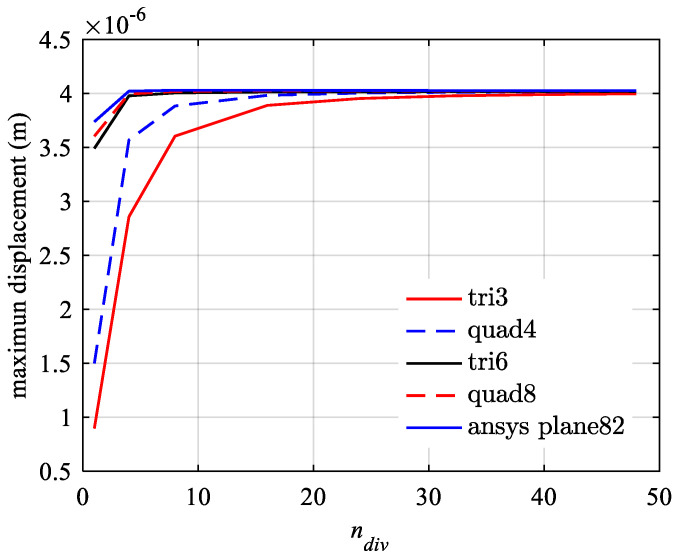
Validation of the present model with the ANSYS plane82 element.

First, the model presented in this paper is degenerated to a tension–compression isotropic modulus model and verified using the commercial software ANSYS 2022 R1 with the plane82 element, employing the same mesh size. The material parameters are set to $E_{t}=E_{c}=200\mathrm{GPa},\;v_{t}=v_{c}=0.3$. The maximum displacement verification results under load F/b=20,000 N/m are shown in Figure 10 and Table 1. It can be observed that as the number of elements on one side increases (i.e., the mesh becomes denser), the calculated results for the maximum displacement approach the true value. The QUAD8 element achieves convergence with a relatively sparse mesh, while the TRI3 element requires a denser mesh to achieve convergence. The order of mesh density requirements for achieving the same accuracy level is QUAD8 < TRI6 < QUAD4 < TRI3. Additionally, Figure 10 also verifies the mesh independence.

#### 3.2.2. Verification with Other Methods of Different Moduli in Tensile and Compressive

**Figure 11:**
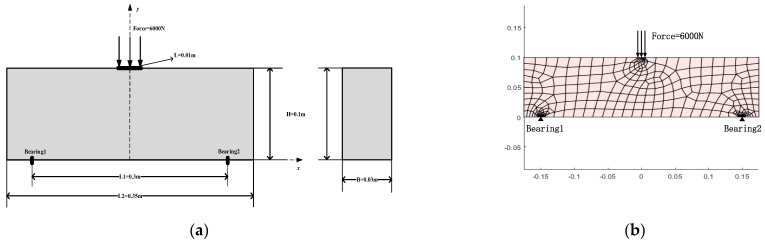
Bending test model constructed for comparison purposes. (**a**) Schematic diagram of the three-point bending test model, (**b**) diagram of the 4-node quadrilateral element mesh model.

To verify the accuracy and reliability of the finite element model considering the bimodulus property presented in this paper, a three-point bending test model (TPB beam) was constructed according to the description in the literature [50], and a detailed comparative analysis was carried out. As shown in Figure 11a, the structural model has the following characteristics: the distance between the two lower supports $L_{1}$ is 300 mm, the total length of the model $L_{2}$ is 350 mm, the height of the model H is 100 mm, and the thickness of the material B is 30 mm. On the top of the model, a vertical downward force of 6000 N was applied through the loading head, with the loading in contact with the model for a length L of 10 mm. In the coordinate system, the x-axis represents the horizontal direction, and the y-axis represents the vertical direction. Four different methods presented in this paper were used to construct the simulation model: 3-node triangular elements, 4-node quadrilateral elements, 6-node triangular elements, and 8-node quadrilateral elements. Among them, the 4-node quadrilateral element model and its boundary conditions are displayed in Figure 11b for observation and understanding. For simplicity, the detailed settings of other models are not shown one by one here.

**Figure 12:**
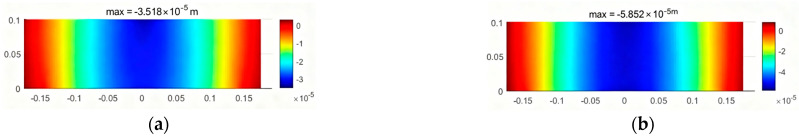
Vertical displacement contour of bending test model. (**a**) Vertical displacement contour considering tension–compression isotropic modulus, (**b**) vertical displacement contour considering bimodulus property.

Under the condition of tension–compression isotropic modulus, the material parameters we set are $E_{t}=E_{c}=60\;\mathrm{GPa}$, $v_{t}=v_{c}=0.25$; while under the condition of tension–compression anisotropic modulus, the material parameters are $E_{t}=20\;\mathrm{GPa}$, $E_{c}=60\;\mathrm{GPa}$, $v_{t}=0.1$, $v_{c}=0.3$ (Conforming to the theoretical assumption in this paper: $E_{t}\nu_{c}=E_{c}\nu_{t}$). Figure 12a shows the vertical displacement contour of the tension–compression isotropic modulus model simulated using 4-node quadrilateral elements, and Figure 12b shows the vertical displacement contour of the tension–compression anisotropic modulus model using the same type of elements. The displacement contours intuitively display that the displacement distribution calculated by this algorithm is highly consistent with the results in the literature [50]. Furthermore, Table 2 lists in detail the calculation results of the different element models presented in this paper and compares them with the results in the literature [50]. These results verify the accuracy and reliability of the model presented in this paper.

## 4. Results and Discussion

**Figure 13:**
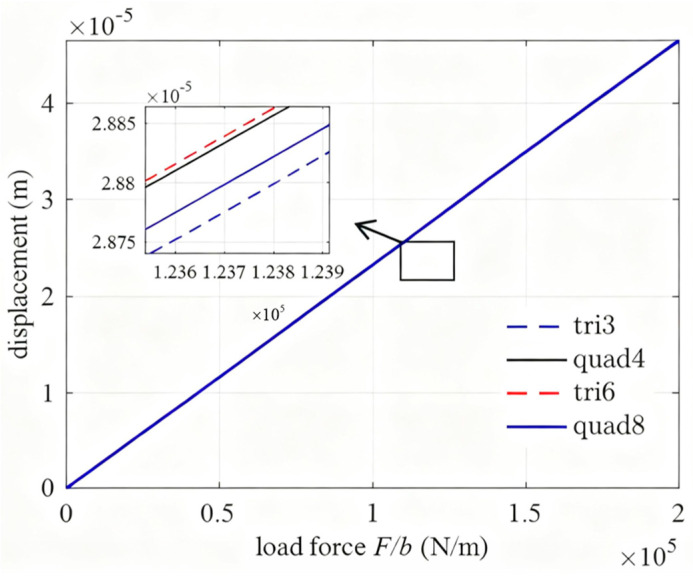
Displacement vs. load force of bimodulus materials.

Considering the structure shown in Figure 8, with parameter settings of $E_{t}=200\;\mathrm{GPa},\;E_{c}=150\;\mathrm{GPa},\;v_{t}=0.3,\;v_{c}=0.225$, a length L of 0.2 m, and a height h of 0.1 m, the load–displacement curve is examined. To ensure solution accuracy, different meshes are used for different elements. According to the results in Table 1, for the TRI3 element, $n_{div}=40$, for the QUAD4 element, $n_{div}=24$, for the TRI6 element, $n_{div}=8$, and for the QUAD8 element, $n_{div}=4$. Such settings are adopted to control the calculation result errors of each model within 1% or less. The calculation results are shown in Figure 13. It can be observed that the results calculated by the four models have good consistency. What differs is the size of the element mesh required to achieve the same accuracy, which can be selected as needed when solving actual problems. Additionally, although the material is considered to have a bimodulus characteristic and exhibits nonlinearity, for the same type of load, the displacement–load curve remains linear. This holds true for cases of proportional loading where the distributions of tensile and compressive regions remain unchanged, whereas it does not apply to scenarios involving mixed boundary conditions or situations where increased loading alters the distribution of tensile and compressive zones. This is because as the load increases, the internal stress in the structure also increases proportionally. Changing only the load magnitude does not change the principal stress direction within the structure, thus not causing changes in the tension–compression regions, affecting the overall stiffness of the structure.

**Figure 14:**
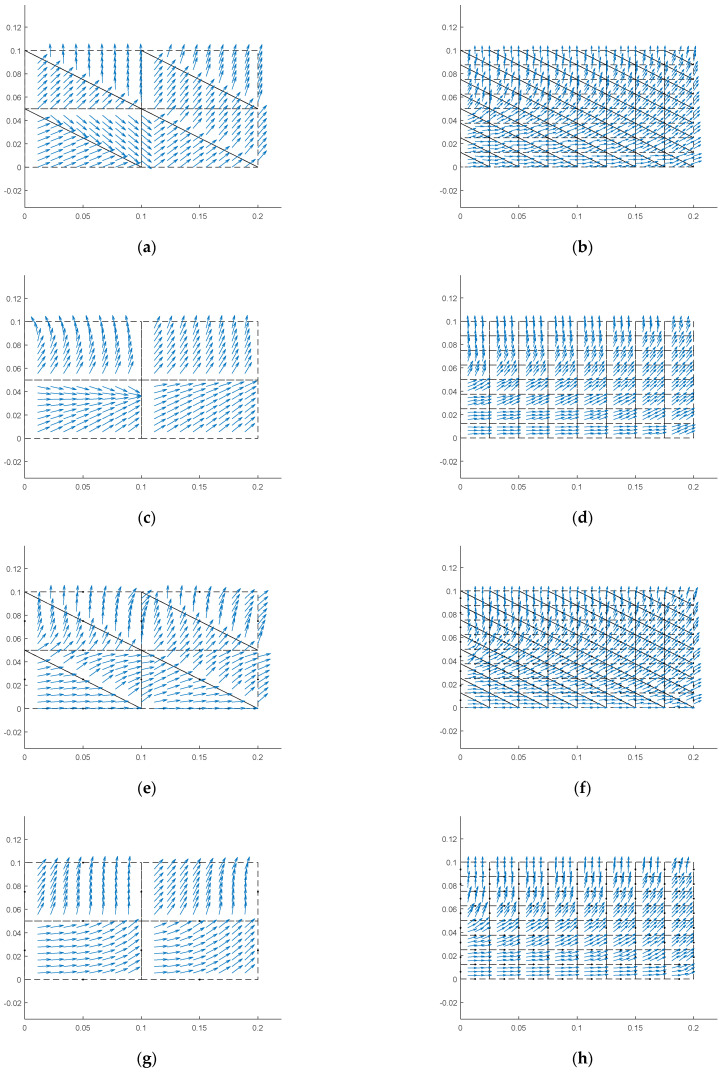
Orientation of the maximum principal stress within various element models. (**a**) tri3 element with ndiv = 2, (**b**) tri3 element with ndiv = 8, (**c**) quad4 element with ndiv = 2, (**d**) quad4 element with ndiv = 8, (**e**) tri6 element with ndiv = 2, (**f**) tri6 element with ndiv = 8, (**g**) quad8 element with ndiv = 2, (**h**) quad8 element with ndiv = 8.

Figure 14 displays the orientation of the maximum principal stress within various element models. It is evident that within a single TRI3 element, the principal stress directions are uniform, while the QUAD4, TRI6, and QUAD8 elements are capable of simulating variations in the principal stress directions within the element. The capacity of different element types to simulate the principal stress directions is contingent upon Equations (57), (60), (63) and (65). The outcomes depicted in the figure suggest that higher-order elements possess enhanced capabilities for simulating principal stress directions. Despite the inherent differences in the simulation capabilities among the elements, employing a finer meshing technique can mitigate these differences.

**Figure 15:**
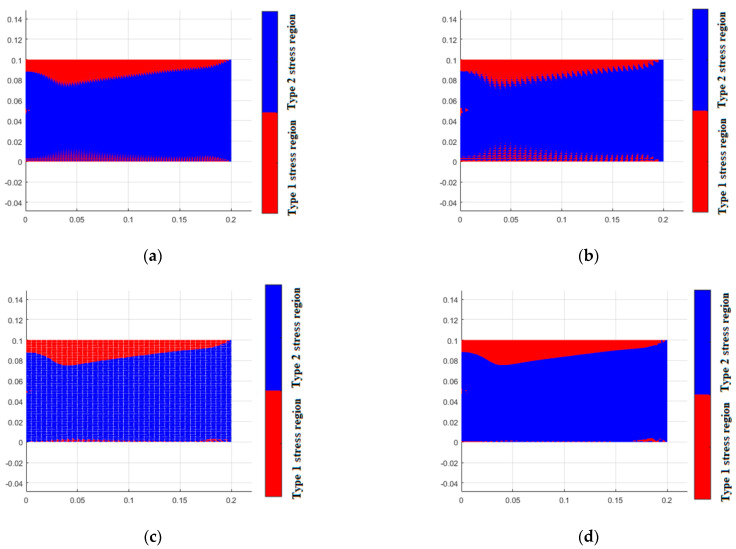
Types of stress region for different element models (Et = 200 GPa, Ec = 180 GPa). (**a**) tri3, (**b**) quad4, (**c**) tri6, (**d**) quad8.

**Figure 16:**
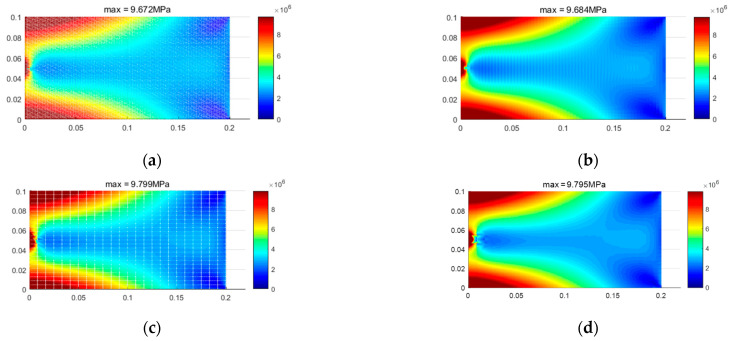
Stress distribution for different element models (Et = 200 GPa, Ec = 180 GPa). (**a**) tri3, (**b**) quad4, (**c**) tri6, (**d**) quad8.

As previously mentioned, when considering the bimodulus characteristics, the stress state within the material is divided into two types of regions. In the first type of region, the material properties in both principal stress directions are identical, and there is no difference between the different modulus theory and classical elasticity theory. In the second type of region, the material properties in the two principal stress directions are different. Figure 15 shows the simulation of these two types of regions by different element models. It can be seen that the results from TRI3 and QUAD4 elements exhibit an unclear interface between the two regions, and even some simulation distortion occurs, with the two types of regions intermingling, which is clearly inconsistent with the actual situation. A finer mesh is required to achieve more accurate results. In contrast, TRI6 and QUAD8 elements can obtain a clear interface and relatively accurate results even with fewer element meshes. Figure 16 displays the stress calculation results for different element models, and the equivalent stress results of the four-element models show a high degree of consistency. At the same time, TRI6 and QUAD8 elements demonstrate comparable accuracy and higher consistency than TRI3 and QUAD4 elements. Therefore, in practical problems where there is a high precision requirement for stress examination, TRI6 and QUAD8 elements should be given priority for simulation.

**Figure 17:**
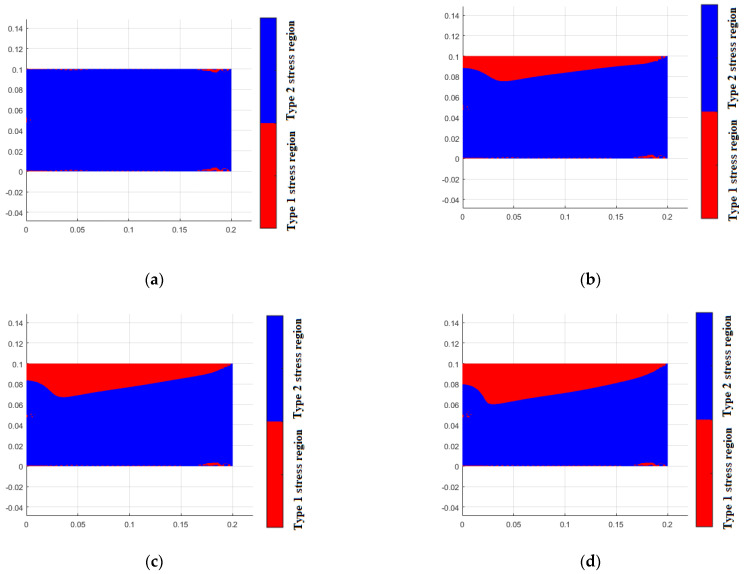

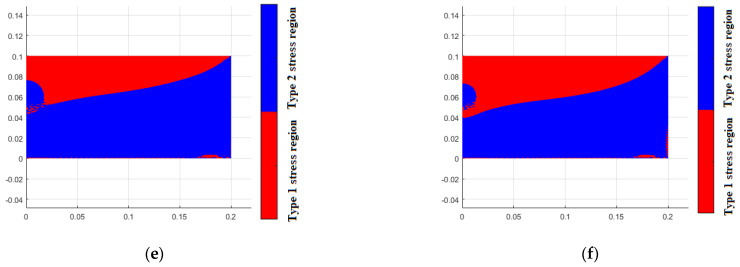
Stress region for different Et/Ec. (**a**) Et = 200 GPa, Ec = 200 GPa, (**b**) Et = 200 GPa, Ec = 180 GPa, (**c**) Et = 200 GPa, Ec = 160 GPa, (**d**) Et = 200 GPa, Ec = 140 GPa, (**e**) Et = 200 GPa, Ec = 120 GPa, (**f**) Et = 200 GPa, Ec = 100 GPa.

**Figure 18:**
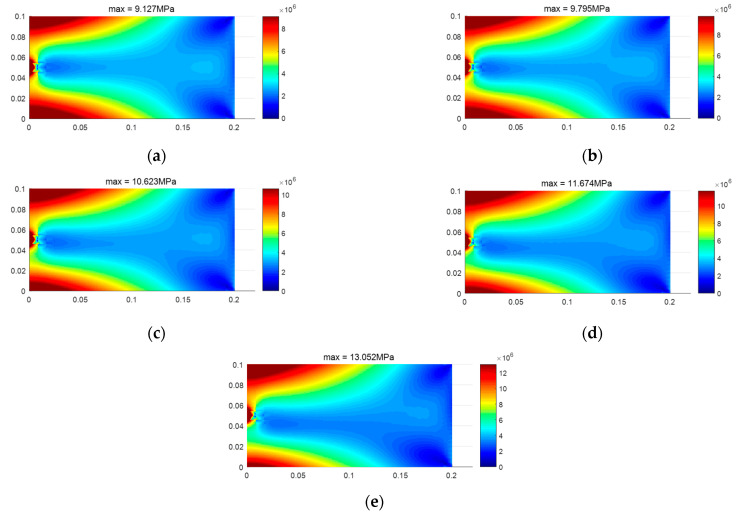
Stress distribution for different Et/Ec. (**a**) Et = 200 GPa, Ec = 200 GPa, (**b**) Et = 200 GPa, Ec = 180 GPa, (**c**) Et = 200 GPa, Ec = 160 GPa, (**d**) Et = 200 GPa, Ec = 140 GPa, (**e**) Et = 200 GPa, Ec = 120 GPa.

Figure 17 illustrates the distribution of the two types of stress regions at different tension–compression modulus ratios, while Figure 18 shows the stress distribution at varying tension–compression modulus ratios. The figures demonstrate the significant impact of the bimodulus beam characteristics on the distribution of stress regions and the maximum stress values. These results validate the effectiveness of the applied constitutive model and finite element framework in capturing the mechanical behavior of bimodulus materials under varying modulus ratios.

**Figure 19:**
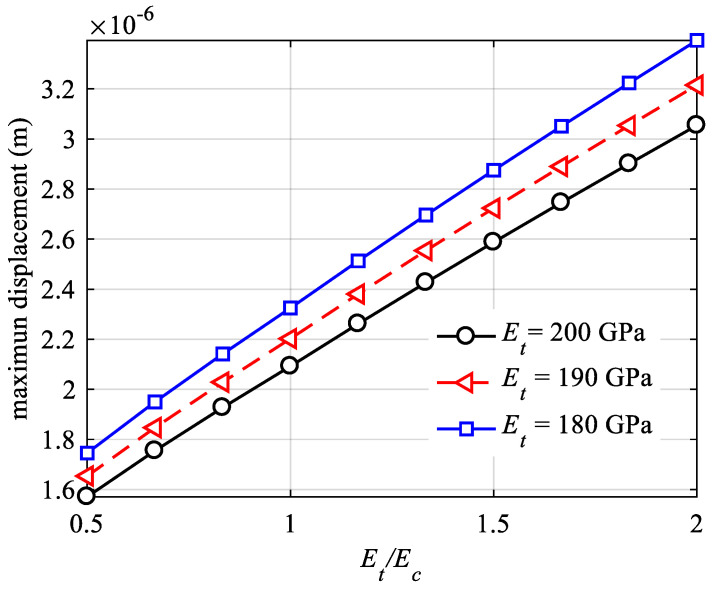
Maximum displacement for different Young’s moduli.

**Figure 20:**
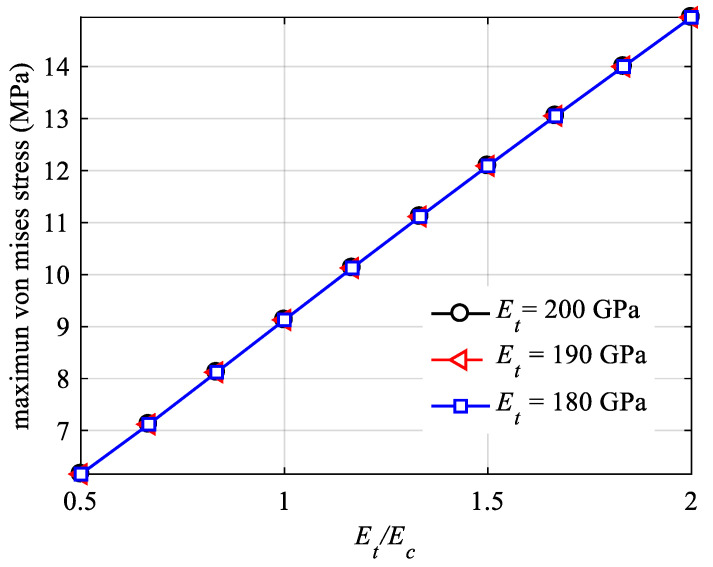
Maximum von Mises stress for different Young’s moduli.

Figure 19 presents the maximum displacement versus tension–compression modulus ratio curve, and Figure 20 illustrates the maximum von Mises stress versus tension–compression modulus ratio curve. The curves in both figures are plotted with a fixed tensile modulus $E_{t}$, examining the outcomes as the tension–compression modulus ratio $E_{t}/E_{c}$ varies. It is evident that the tension–compression modulus ratio $E_{t}/E_{c}$ significantly influences both displacement and stress. In Figure 19, different maximum displacement results for the same tension–compression modulus ratio $E_{t}/E_{c}$ are observed due to the varying overall equivalent stiffness of the structure. Conversely, in Figure 20, the maximum von Mises stress results remain consistent for different values of the tension–compression modulus ratio $E_{t}/E_{c}$ under the same $E_{t}$, indicating that when the body force is zero, the stress distribution is related to the load, boundary conditions, and the structure itself, rather than the material properties. This finding aligns with the conclusions of the theory of equal tension–compression modulus (classical elasticity theory).

**Figure 21:**
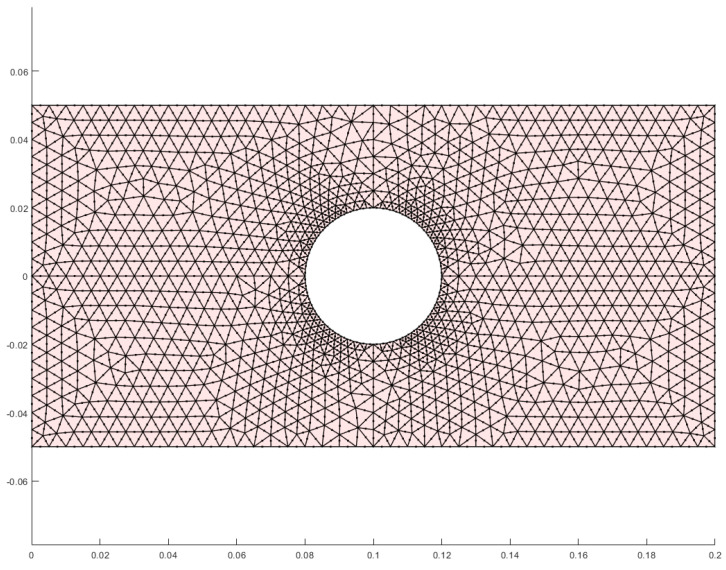
Finite element model of a square plate with a central circular hole and different tension–compression moduli.

**Figure 22:**
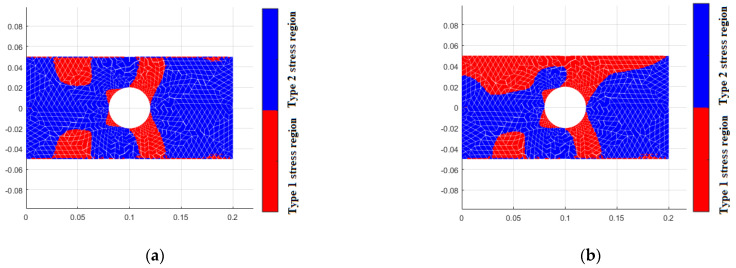
Distribution of two types of stress regions. (**a**) Et = 200 GPa, Ec = 200 GPa, (**b**) Et = 200 GPa, Ec = 150 GPa.

**Figure 23:**
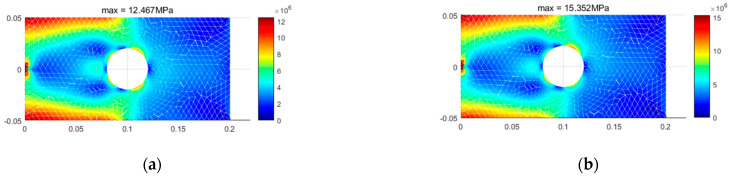
Stress contour of the overall structure. (**a**) Et = 200 GPa, Ec = 200 GPa, (**b**) Et = 200 GPa, Ec = 150 GPa.

**Figure 24:**
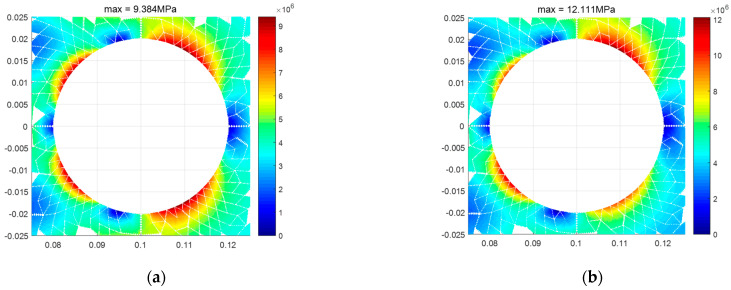
Stress contour at the edge of the hole. (**a**) Et = 200 GPa, Ec = 200 GPa, (**b**) Et = 200 GPa, Ec = 150 GPa.

To further investigate the applicability of the finite element theory presented in this paper, a square plate with a center circular hole and different tension–compression moduli is examined. Given the high precision of the TRI6 element and its strong capability in simulating the principal stress directions, along with its adaptability to complex structures, calculations are performed using the TRI6 element. After meshing the structure, the finite element model is obtained as shown in Figure 21. Figure 22 displays the results of the two types of stress regions, Figure 23 shows the stress contour of the overall structure, and Figure 24 illustrates the stress contour at the edge of the hole. The results demonstrate that after accounting for the difference between tensile and compressive moduli, the types of stress zones undergo significant changes (Figure 22). Moreover, the maximum stress at the hole edge increases from 9.36 MPa to 12.11 MPa (Figure 24). The complex stress state around the hole, where the principal stress directions significantly deviate from the geometric coordinate axes, presents an ideal scenario for evaluating the performance of the non-principal-direction constitutive model.

## 5. Conclusions

This paper systematically applied the constitutive theory of bimodular materials based on principal stresses and combined it with the finite element method to analyze the mechanical behavior of materials with different tensile and compressive moduli. The validation of Ambartsumyan’s theory against ANSYS simulations further confirmed its effectiveness and reliability in numerical simulations, particularly when using higher-order elements such as TRI6 and QUAD8. The iterative solution method adopted in this study effectively addressed the nonlinearity introduced by bimodulus materials and achieved model convergence.

The findings highlight the importance of the tension-to-compression modulus ratio on structural displacement and stress distribution, emphasizing its practical significance in engineering design and material selection. The established finite element models effectively simulated structural responses under complex geometries and loading conditions, demonstrating good engineering applicability. The methods proposed in this study provide practical numerical tools for subsequent research, facilitating further investigations into complex working conditions such as multiaxial stress states, material degradation, and fatigue.

The finite element models developed in this study serve as effective numerical tools for the structural analysis and design of bimodulus materials, contributing to improved simulation accuracy and design reliability in engineering applications. This work holds reference value for promoting the application of bimodulus materials in engineering structures.

## Figures and Tables

**Table 1:** Maximum displacements (μm) and errors (refer to the result of the ANSYS plane82 element model with $n_{div}=48$) of different models.

$n_{div}$	Tri3	Error	Quad4	Error	Tri6	Error	Quad8	Error	Plane82	Error
1	0.8970	−77.72%	1.4998	−62.75%	3.4915	−13.29%	3.6047	−10.48%	3.7389	−7.14%
4	2.8582	−29.02%	3.5700	−11.34%	3.9786	−1.19%	3.9963	−0.75%	4.0219	−0.11%
8	3.6056	−10.45%	3.8848	−3.52%	4.0058	−0.52%	4.0189	−0.19%	4.0305	0.10%
16	3.8903	−3.38%	3.9833	−1.07%	4.0149	−0.29%	4.0246	−0.05%	4.0299	0.08%
24	3.9541	−1.80%	4.0044	−0.55%	4.0166	−0.25%	4.0252	−0.03%	4.0287	0.05%
32	3.9786	−1.19%	4.0124	−0.35%	4.0171	−0.23%	4.0252	−0.03%	4.0278	0.03%
40	3.9907	−0.89%	4.0163	−0.25%	4.0173	−0.23%	4.0251	−0.04%	4.0271	0.01%
48	3.9976	−0.72%	4.0185	−0.20%	4.0173	−0.23%	4.0249	−0.04%	4.0265	0.00%

**Table 2:** Comparison of the present model with available results.

	Ref [50]	Present							
		Tri3		Quad4		Tri6		Quad8	
			Error		Error		Error		Error
Same Modulus	3.5 × 10^−5^	3.432 × 10^−5^	1.95%	3.518 × 10^−5^	0.51%	3.734 × 10^−5^	6.69%	3.712 × 10^−5^	6.05%
Different Modulus	6 × 10^−5^	5.710 × 10^−5^	4.83%	5.852 × 10^−5^	2.46%	6.136 × 10^−5^	2.27%	6.114 × 10^−5^	1.9%

## Data Availability

The original contributions presented in this study are included in the article. Further inquiries can be directed to the corresponding author.

## References

[1] Beskopylny A., Meskhi B., Kadomtseva E., Strelnikov G. (2020). Transverse Impact on Rectangular Metal and Reinforced Concrete Beams Taking into Account Bimodularity of the Material. Materials.

[2] Wang D., Xu H., Sun P. (2023). Performance analysis of bimodular simply supported beams based on the deformation decomposition method. Mech. Adv. Mater. Struct..

[3] Zhang Z., Yao C., Yu Y., Hong Z., Zhi M., Wang X. (2016). Mesoporous piezoelectric polymer composite films with tunable mechanical modulus for harvesting energy from liquid pressure fluctuation. Adv. Funct. Mater..

[4] Viet N.V., Zaki W., Umer R. (2020). Analytical investigation of an energy harvesting shape memory alloy–piezoelectric beam. Arch. Appl. Mech..

[5] Wang J., Xiao Y., Inoue K., Kawai M., Xue Y. (2019). Modeling of nonlinear response in loading-unloading tests for fibrous composites under tension and compression. Compos. Struct..

[6] Barak M.M., Currey J.D., Weiner S., Shahar R. (2009). Are tensile and compressive Young’s moduli of compact bone different?. J. Mech. Behav. Biomed. Mater..

[7] Xu S., Liu J., Li X., Ma Y. (2022). A full-scale topology optimization method for surface fiber reinforced additive manufacturing parts. Comput. Methods Appl. Mech. Eng..

[8] Bert C.W. (1977). Models for fibrous composites with different properties in tension and compression. J. Eng. Mater. Technol..

[9] Destrade M., Gilchrist M.D., Motherway J., Murphy J. (2010). Bimodular rubber buckles early in bending. Mech. Mater..

[10] Pastor-Artigues M.-M., Roure-Fernández F., Ayneto-Gubert X., Bonada-Bo J., Pérez-Guindal E., Buj-Corral I. (2019). Elastic Asymmetry of PLA Material in FDM-Printed Parts: Considerations Concerning Experimental Characterisation for Use in Numerical Simulations. Materials.

[11] Jones R.M. (2012). Stress-strain relations for materials with different moduli in tension and compression. AIAA J..

[12] Ambartsumyan S.A. (1982). Different Modulus Theory of Elasticity.

[13] He X.-T., Pei X.-X., Sun J.-Y., Zheng Z.-L. (2016). Simplified theory and analytical solution for functionally graded thin plates with different moduli in tension and compression. Mech. Res. Commun..

[14] Oppermann R.H. (1941). Strength of materials, part 2, advanced theory and problems: By S. Timoshenko, second edition. J. Frankl. Inst..

[15] Jones R.M. (1976). Apparent Flexural Modulus and Strength of Multimodulus Materials. J. Compos. Mater..

[16] Bert C., Gordaninejad F. (1983). Transverse shear effects in bimodular composite laminates. J. Compos. Mater..

[17] Ambartsumyan S.A., Wu R.F., Zhang Y.Z. (1986). Elasticity Theory of Different Moduli.

[18] Reddy J.N., Chao W.C. (1983). Nonlinear bending of bimodular-material plates. Int. J. Solids Struct..

[19] Zinno R., Greco F. (2001). Damage evolution in bimodular laminated composites under cyclic loading. Compos. Struct..

[20] Chen L.-W., Chen C.C. (1989). Asymmetric vibration and dynamic stability of bimodulus thick annular plates. Comput. Struct..

[21] Li X., Sun J.-Y., Dong J., He X.-T. (2018). One-Dimensional and Two-Dimensional Analytical Solutions for Functionally Graded Beams with Different Moduli in Tension and Compression. Materials.

[22] He X.-T., Li W.-M., Sun J.-Y., Wang Z.-X. (2018). An elasticity solution of functionally graded beams with different moduli in tension and compression. Mech. Adv. Mater. Struct..

[23] Chao G. (1997). Analysis for the Canopy of Plane with the Theory of Extension Compression Elastic Modulus. J. Dalian Fish. Univ..

[24] Chao G., Xianqiang L. (1998). Analysis for the plate with the theory of different extension compression elastic modulis. Chin. J. Comput. Mech..

[25] Gao C., Zhang Y.Z., Lu X.Q. (2000). Analysis of thin-shell structures based on bi-moduli theory. Eng. Mech..

[26] Yan Y., Meng X., Qu C. (2022). Modeling of Bimodular Bone Specimen under Four-Point Bending Fatigue Loading. Materials.

[27] Ye Z.M., Chen T., Yao W.J. (2004). Progresses in elasticity theory with different modulus in tension and compression and related FEM. Mech. Eng..

[28] Ye Z., Wang D., Chen T. (2009). Numerical study for load-carrying capacity of beam-column members having different Young′s moduli in tension and compression. Int. J. Model. Identif. Control.

[29] Yao W.-J., Zhang C.-H., Jiang X.-F. (2006). Nonlinear Mechanical Behavior of Combined Members with Different Moduli. Int. J. Nonlinear Sci. Numer. Simul..

[30] Rong X., Zheng J., Jiang C. (2023). Topology optimization for structures with bi-modulus material properties considering displacement constraints. Comput. Struct..

[31] Latorre M., Montáns F.J. (2020). Bi-modulus materials consistent with a stored energy function: Theory and numerical implementation. Comput. Struct..

[32] Du Z., Zhang Y., Zhang W., Guo X. (2016). A new computational framework for materials with different mechanical responses in tension and compression and its applications. Int. J. Solids Struct..

[33] Rebecca G., Giulia M., Luisa R. (2023). A Bi-Modulus Material Model for Bending Test on NHL3.5 Lime Mortar. Materials.

[34] Pan Q.X., Zheng J.L., Li Q., Wen P.H. (2020). Fracture analysis for bi-modular materials. Eur. J. Mech. A/Solids.

[35] He X.-T., Wang X.-G., Sun J.-Y. (2024). Application of perturbation-variation method in large deformation bimodular cylindrical shells: A comparative study of bending theory and membrane theory. Appl. Math. Model..

[36] Ren X., Du Z., Chung H., Tang S., Guo Y., Chen B., Guo X. (2024). Finite deformation analysis of bi-modulus thermoelastic structures and its application in wrinkling prediction of membranes. Comput. Methods Appl. Mech. Eng..

[37] Zhao H., Ye Z. (2015). Analytic elasticity solution of bi-modulus beams under combined loads. Appl. Math. Mech..

[38] Medri G. (1982). A Nonlinear Elastic Model for Isotropic Materials with Different Behavior in Tension and Compression. J. Eng. Mater. Technol..

[39] Ambartsumyan S.A., Khachatryan A.A. (1966). The basic equations of the theory of elasticity for materials with different tensile and compressive stiffness. Russ. Electr. Eng..

[40] Yunzhen Z., Zhifeng W. (1989). The finite element method for elasticity with different moduli in tension and compression. Comput. Struct. Mech. Appl..

[41] He X.T., Zheng Z.L., Sun J.Y., Li Y.M., Chen S.L. (2009). Convergence analysis of a finite element method based on different moduli in tension and compression. Int. J. Solids Struct..

[42] Shan-Lin C. (2008). Approximate Elasticity Solution of Bending-compression Column with Different Tension-compression Moduli. J. Chongqing Univ..

[43] Xie W.H., Peng Z.J., Meng S.H., Xu C.H., Yi F.J., Du S.Y. (2016). GWFMM model for bi-modulus orthotropic materials: Application to mechanical analysis of 4D-C/C composites. Compos. Struct..

[44] Yang H., Wang B. (2008). An analysis of longitudinal vibration of bimodular rod via smoothing function approach. J. Sound Vib..

[45] Zhang L., Gao Q., Zhang H.W. (2013). An efficient algorithm for mechanical analysis of bimodular truss and tensegrity structures. Int. J. Mech. Sci..

[46] Pan Q., Zheng C., Song X., Lv S., Yu H., Zhang J., Cabrera M.B., Liu H. (2021). Mechanical analysis of asphalt pavement based on bimodulus elasticity theory. Constr. Build. Mater..

[47] Iandiorio C., Serenella R., Salvini P. (2025). A Combined Approach of Experimental Testing and Inverse FE Modelling for Determining Homogenized Elastic Properties of Membranes and Plates. Eng. Proc..

[48] Huang T., Pan Q., Jin J., Zheng J., Wen P. (2018). Continuous constitutive model for bimodulus materials with meshless approach. Appl. Math. Model..

[49] Zhou Y., Yang L., Huang Y. (2013). Micro- and Macromechanical Properties of Materials.

[50] Wang T., Ye J. (2023). Numerical analysis of bending property of bi-modulus materials and a new method for measurement of tensile elastic modulus. J. Rock Mech. Geotech. Eng..

